# Molybdenum(0) tricarbonyl and tetracarbonyl complexes with a cationic pyrazolylpyridine ligand: synthesis, crystal structures and catalytic performance in olefin epoxidation†

**DOI:** 10.1039/c8ra01687a

**Published:** 2018-05-01

**Authors:** Lucie S. Nogueira, Patrícia Neves, Ana C. Gomes, Pedro Lavrador, Luís Cunha-Silva, Anabela A. Valente, Isabel S. Gonçalves, Martyn Pillinger

**Affiliations:** Department of Chemistry, CICECO – Aveiro Institute of Materials, University of Aveiro, Campus Universitário de Santiago 3810-193 Aveiro Portugal mpillinger@ua.pt,atav@ua.pt; REQUIMTE/LAQV, Department of Chemistry and Biochemistry, Faculty of Sciences, University of Porto 4169-007 Porto Portugal

## Abstract

The synthesis of molybdenum(0) tricarbonyl and tetracarbonyl complexes of the form [Mo(CO)_3_(ptapzpy)Br] (1) and *cis*-[Mo(CO)_4_(ptapzpy)]Br (2) is reported, where ptapzpy = 2-(1-propyltrimethylammonium-3-pyrazolyl)pyridine. Preparation of these derivatives was accomplished either through thermal replacement of CO in Mo(CO)_6_ (for 1) or substitution under milder conditions of piperidine ligands in the precursor *cis*-[Mo(CO)_4_(pip)_2_] (for 2). The crystal structures of the ligand [ptapzpy]Br and complexes 1 and 2 were determined. Thermal treatment of 2 at 125–150 °C leads to mono decarbonylation and formation of 1. On the other hand, oxidative decarbonylation of 1 and 2 by reaction with *tert*-butylhydroperoxide (TBHP, 10 equiv.) gives a molybdenum oxide hybrid material formulated as [Mo_3_O_9_([ptapzpy]Br)_2_]·nH_2_O (3), which was characterised by FT-IR and Raman spectroscopy, thermogravimetric analysis, and ^13^C{^1^H} CP MAS NMR spectroscopy. Compounds 1–3 were effective (pre)catalysts for the epoxidation of *cis*-cyclooctene at 55 °C with aqueous H_2_O_2_ or TBHP (slightly better results were obtained with the former). The characterisation of the Mo-containing solids isolated after the catalytic reaction showed that poorly soluble β-octamolybdate salts, (L)_*x*_[Mo_8_O_26_], were formed from 1–3 with TBHP and from 1 with H_2_O_2_, while soluble oxoperoxo species were formed from 3 with H_2_O_2_. These findings helped to explain the different catalytic performances obtained.

## Abstract 2

The oxidative decarbonylation and catalytic chemistry of molybdenum(0) tricarbonyl and tetracarbonyl complexes containing the same diimine ligand are compared.

## Graphical abstract

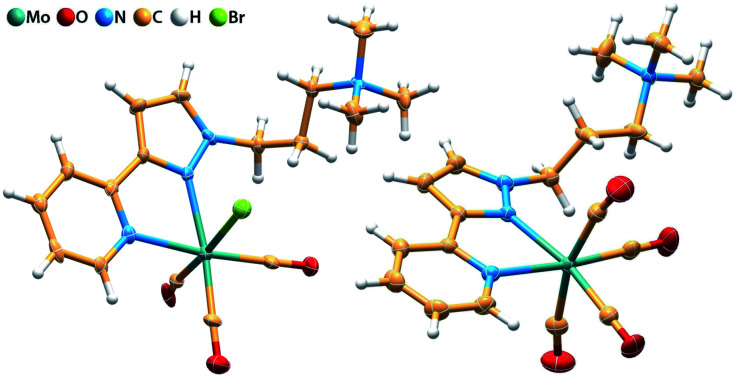

## Introduction

The organometallic chemistry of molybdenum dates back to the mid 1930s when Hieber and co-workers described the thermal substitution of CO ligands in Mo(CO)_6_ to give octahedral Mo^0^ derivatives of the type Mo(CO)_*x*_(L)_*y*_.^1^ The physical properties of one of these compounds, *cis*-[Mo(CO)_4_(bipy)] (bipy = 2,2′-bipyridine), were investigated much later by Stiddard,^2^ and the molecular structure of this complex was determined only recently.^3^ Since the report by Stiddard, much attention has been given to molybdenum(0)-diimine-tetracarbonyl complexes containing bidentate ligands (N–N) such as bipyridines,^3,4^ phenanthrolines,^5^ 1,4-diazabutadienes,^6^ iminopyridines,^7^ and pyrazolylpyridines.^8^ Further substitution of the carbonyl ligands is possible (without alteration of the formal charge on the central Mo atom), *e.g.* of one carbonyl ligand by a neutral (L) or anionic coligand (X^−^) to give tricarbonyl complexes [Mo(CO)_3_(N–N)(L/X)]^0/−^,^6*a*,9^ or of two carbonyl ligands by a second diimine ligand to give dicarbonyl complexes [Mo(CO)_2_(N–N)_2_].^10^ Although anionic complexes [Mo(CO)_3_(N–N)(X)]^−^ were described by Behrens *et al.* in 1970,^9*a*^ molecular structures for this family of compounds were only described six years ago.^6*a*^

Complexes of the type [Mo(CO)_3_(N–N)(X)]^−^ can be prepared from *cis*-[Mo(CO)_4_(N–N)]^9*b*^ but not in the reverse sense from the pentacarbonyl complexes [Mo(CO)_5_(X)]^−^, since reaction of the latter with the ligand N–N gives instead the tetracarbonyl derivatives *cis*-[Mo(CO)_4_(N–N)].^11^ This was attributed to preferential halogen displacement (rather than CO substitution) in the intermediate [Mo(CO)_4_(N–N)(X)]^−^ owing to the fact that bidentate diimines such as bipy are relatively poor π-acceptors, thereby strengthening the remaining Mo–C bonds in the intermediate.^11^ To the best of our knowledge, the direct synthesis of anionic complexes [Mo(CO)_3_(N–N)(X)]^−^ from molybdenum hexacarbonyl has never been reported.

Carbonyl–diimine complexes of Mo^0^, especially the tetracarbonyl derivatives, have been intensively studied due to their rich electronic absorption spectra, photophysics, and photochemistry.^12^ Tetracarbonyl derivatives are strongly colored due to an intense Mo → diimine metal-to-ligand charge-transfer (MLCT) absorption band in the visible spectral region. The low-lying MLCT excited state is associated with several interesting phenomena such as negative solvatochromism,^6*a*,12*c*–*e*^ second-order nonlinear optical responses,^12*f*,*g*^ luminescence,^12*a*–*c*^ and photochemically induced CO substitution.^12*a*,*h*,*i*^ Aside from their photo-properties, Mo carbonyl diimine complexes possess other interesting features and have been studied as CO-releasing molecules for therapeutic applications,^13*a*^ catalysts for the electrochemical reduction of CO_2_,^13*b*–*d*^ initiators for the polymerisation of methyl methacrylate,^13*e*^ alkylation of allylic acetates,^5*b*^ and precatalysts for the epoxidation of olefins.^4*a*,8^ In the latter case, the catalyst precursors undergo oxidative decarbonylation (OD) by reaction with an oxidant to give oxomolybdenum(vi)-diimine compounds, the structures of which vary according to the nature of the organic ligand. With tetracarbonyl precursors, structurally characterised OD products include tetranuclear [Mo_4_O_12_(pzpy)_4_] (pzpy = 2-[3(5)-pyrazolyl]pyridine),^8*a*^ octanuclear [Mo_8_O_24_(di-*t*Bu-bipy)_4_] (di-*t*Bu-bipy = 4,4′-di-*tert*-butyl-2,2′-bipyridine),^4*a*^ and the one-dimensional (1D) molybdenum oxide/bipyridine polymer [MoO_3_(bipy)].^4*a*^

As part of our continuing interest in pyrazolylpyridine ligands, we have prepared the cationic derivative 2-(1-propyltrimethylammonium-3-pyrazolyl)pyridine bromide ([ptapzpy]Br). The introduction of cation-bearing ligands into transition metal complexes can help improve solubility in polar solvents or target the complexes for immobilisation in solid supports with charged frameworks and/or functional groups. The reaction of [ptapzpy]Br with Mo(CO)_6_ unexpectedly yielded the charge-neutral zwitterionic complex [Mo(CO)_3_(ptapzpy)Br] rather than the tetracarbonyl derivative *cis*-[Mo(CO)_4_(ptapzpy)]Br. For comparison, the latter complex was prepared by a different route and the molecular structures of both complexes have been determined. OD of the complexes with *tert*-butylhydroperoxide (TBHP) is shown to give a hybrid molybdenum oxide/organic material. The catalytic behavior and chemical transformations of all compounds in the epoxidation of *cis*-cyclooctene have been studied using TBHP or H_2_O_2_ as oxidant.

## Results and discussion

### Synthesis and characterisation

**Scheme 1:**
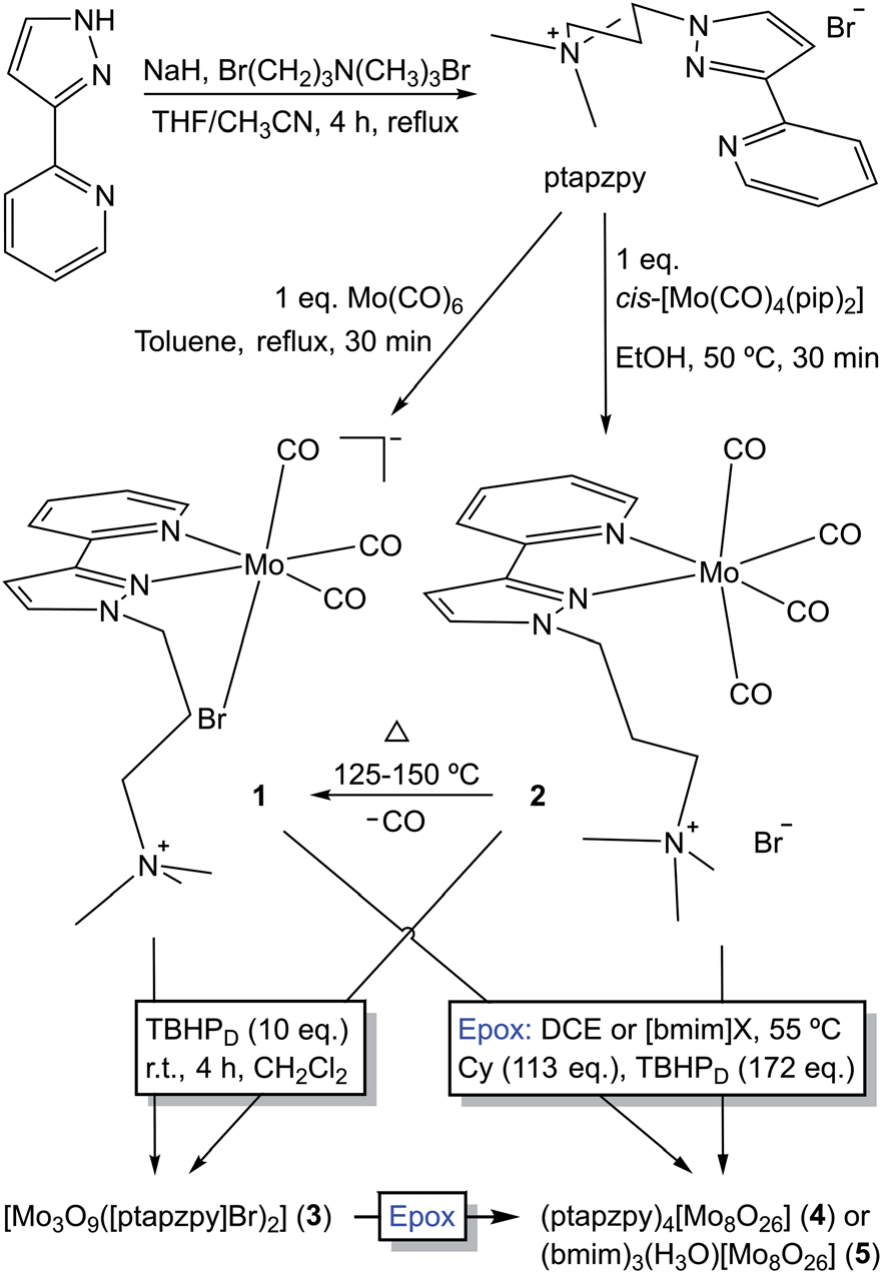
Preparation of [ptapzpy]Br, complexes 1 and 2, and the oxidised compounds formed by OD of the carbonyl complexes by reaction with 10 eq. of TBHP or a large excess of TBHP under the catalytic epoxidation reaction conditions (denoted Epox).

The ligand [ptapzpy]Br was prepared by deprotonation of 2-(3-pyrazolyl)pyridine followed by addition of (3-bromopropyl)trimethylammonium bromide and reflux for 4 h (Scheme 1). Characterisation data (FT-IR, ^1^H and ^13^C solution NMR) were in line with those reported previously for related 2-(1-alkyl-3-pyrazolyl)pyridine derivatives.^14^ [ptapzpy]Br is highly hygroscopic, soluble in water, ethanol, acetonitrile, dichloromethane and chloroform, and insoluble in acetone, toluene, 1,2-dichloroethane and diethyl ether.

Reaction of pyrazolylpyridine ligands with 1 equivalent of Mo(CO)_6_ in refluxing toluene usually gives tetracarbonyl complexes of the type *cis*-[Mo(CO)_4_(N–N)].^8^ However, in the case of [ptapzpy]Br, this reaction led to the tricarbonyl derivative [Mo(CO)_3_(ptapzpy)Br] (1) (Scheme 1). The unusual direct synthesis of the tricarbonyl complex from the hexacarbonyl and the ligand results from the presence of the terminal trimethylammonium group in the ligand together with the bromide counterion, which favour the formation of the charge-neutral zwitterionic complex. The tetracarbonyl derivative *cis*-[Mo(CO)_4_(ptapzpy)]Br (2) could be prepared by treatment of the precursor *cis*-[Mo(CO)_4_(pip)_2_] (prepared by the literature method;^15^ pip = piperidine) with 1 equivalent of [ptapzpy]Br in ethanol at 50 °C. Complexes 1 and 2 are at best only sparingly soluble in nonpolar solvents such as chloroform. Dissolution of the compounds in polar, coordinating solvents such as water, acetonitrile and dimethyl sulfoxide is accompanied by solvolysis (especially for 1, as reported previously in ref. 9*b* for other tricarbonyl anions [Mo(CO)_3_(N–N)(X)]^−^) and fast degradation involving decarbonylation. While complex 2 displays extended stability in the solid-state if stored cold in the dark and under inert atmosphere, complex 1 is unstable and undergoes decarbonylation over a period of a few days.

The FT-IR spectra of freshly prepared 1 and 2 confirmed the formation of tricarbonyl and tetracarbonyl complexes, respectively (Fig. S4 in the ESI†). In the carbonyl stretching region (1700–2020 cm^−1^), complex 1 displays two overlapping strong bands at 1742 and 1764 cm^−1^, and one very strong band at 1895 cm^−1^, in a pattern that is typical of *fac*-[Mo(CO)_3_(N–N)(L/X)]^0/−^ complexes.^6*a*,9*b*^ The two lower energy bands arise from splitting of the E mode, while the high-energy band is associated with the A_1_ mode. Four carbonyl stretching bands are observed for complex 2 as expected for *cis*-substituted tetracarbonyl complexes. These are assigned as A^2^_1_ (medium, 2012 cm^−1^), B_1_ (shoulder, 1888 cm^−1^), A^1^_1_ (very strong, 1869 cm^−1^) and B_2_ (very strong, 1815 cm^−1^).^16^ From 300 to 1700 cm^−1^ the vibrational spectra of 1 and 2 exhibit numerous ligand (ptapzpy) modes. Upon complexation the ligand undergoes structural changes which affect the 1550–1650 cm^−1^ region.^8*b*,14*a*,17^ In particular, the pyridyl C–N stretching mode shifts from 1590 cm^−1^ for the free ligand to 1604–1608 cm^−1^ for 1 and 2.

Thermogravimetric analysis (TGA) of 2 revealed a weight loss step of 4.2% between 90 and 125 °C (Fig. S2 in the ESI†). This is close to the calculated value of 5.0% for the removal of one CO ligand. Accordingly, ATR FT-IR spectra (not shown) of 2 heated at different temperatures (15 min at each temperature) showed that the compound is stable at 75 °C but is converted at 125–150 °C to the tricarbonyl complex 1 (Scheme 1). Notably, complex 1 differs from 2 in not displaying a resolved weight loss step around 100 °C. Above 150 °C the TGA curves for 1 and 2 are similar, showing two major decomposition steps in the 200–300 °C and 475–575 °C intervals.

### Single-crystal X-ray structure analyses

**Fig. 1:**
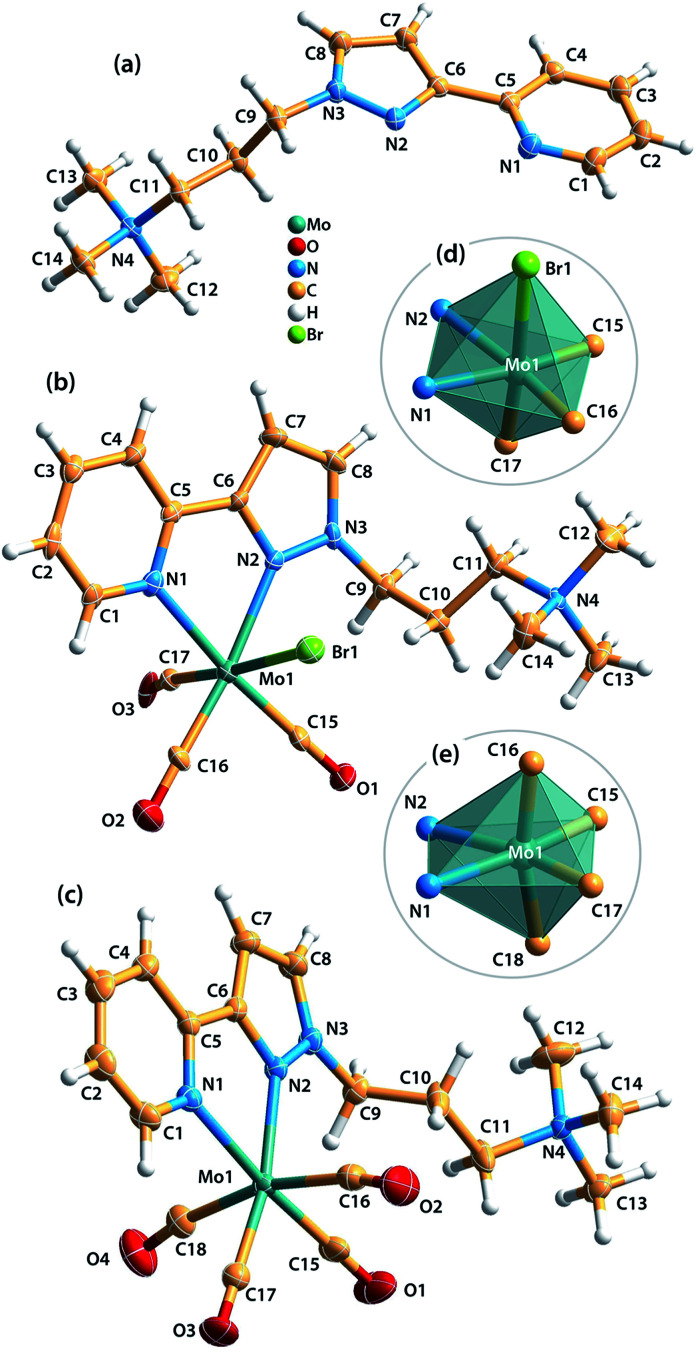
Representations of (a) the molecular structure of the cationic ligand in [ptapzpy]Br, (b) the molecular structure of [Mo(CO)_3_(ptapzpy)Br] in 1·CH_3_CN, (c) the molecular structure of [Mo(CO)_4_(ptapzpy)]^+^ in 2·CH_3_CN, (d) and (e) the Mo^0^ coordination centres found in complexes 1 and 2, respectively. Thermal ellipsoids are drawn at the 50% probability level and the labelling scheme is provided for all non H-atoms. 1·CH_3_CN: selected bond lengths [Å]: Mo1–C15 1.935(6), Mo1–C16 1.894(7), Mo1–C17 2.072(9), Mo1–N1 2.293(5), Mo1–N2 2.259(6), Mo1–Br1 2.7476(10). Selected bond angles [°]: C16–Mo1–C15 85.2(3), N2–Mo1–N1 72.13(19), C17–Mo1–Br1 174.63(19), C15–Mo1–Br1 93.0(2), N1–Mo1–Br1 84.49(13), C17–Mo1–N1 94.6(2). 2·CH_3_CN: selected bond lengths [Å]: Mo1–C15 1.946(2), Mo1–C16 2.042(3), Mo1–C17 1.954(2), Mo1–C18 2.026(3), Mo1–N1 2.2689(17), Mo1–N2 2.2576(17). Selected bond angles [°]: C15–Mo1–C17 89.13(10), N1–Mo1–N2 71.71(6), C18–Mo1–C16 165.75(9), C15–Mo1–C16 86.81(10), C16–Mo1–N1 93.09(8), C18–Mo1–N1 95.96(8).

Single-crystals of [ptapzpy]Br, [Mo(CO)_3_(ptapzpy)Br]·CH_3_CN (1·CH_3_CN) and [Mo(CO)_4_(ptapzpy)]Br·CH_3_CN (2·CH_3_CN) suitable for X-ray diffraction (XRD) were obtained (Table S1 in the ESI†). The cationic organic ligand in [ptapzpy]Br crystallised with the counterion Br^−^ in the monoclinic space group *P*2_1_/*c* (Fig. 1a). In 1, the neutral tricarbonyl complex crystallised with one CH_3_CN molecule of crystallisation in the monoclinic space group *P*2_1_ (Fig. 1b), while in 2 the cationic tetracarbonyl complex crystallised jointly with one Br^−^ counterion and one uncoordinated CH_3_CN molecule in the triclinic space group P1̄ (Fig. 1c).

A striking feature of the crystal structure of [ptapzpy]Br is that the pyrazolylpyridine group adopts a cisoid conformation with respect to the pyridine and pyrazole nitrogen atoms. In analogy with the situation usually encountered with uncoordinated 2,2′-bipyridine derivatives in the solid-state, uncoordinated pyrazolylpyridine ligands typically adopt a transoid conformation, *i.e.* the two rings are rotated along the central C5–C6 bond, thereby minimising repulsion between the lone pairs of the nitrogen atoms and between the protons H-4 and H-8. Crystal packing effects together with a complex network of weak intermolecular interactions (involving the bromide anion) may be responsible for this rare conformation of ptapzpy in the solid-state.

In complex 1, the Mo^0^ centre coordinates to three carbonyl groups, one *N*,*N*-chelating pyrazolylpyridine ligand and one bromide anion, leading to a distorted octahedral coordination geometry with wide ranges for the bond lengths and internal bond angles (Fig. 1d). The bond lengths range from 1.894(7) Å (Mo–C) to 2.7476(10) Å (Mo–Br). Furthermore, the *cis* angles range between 72.13(19) and 101.9(3)°, while the *trans* angles are found in the range of 172.9(3)–174.63(19)°.

The main crystallographic features of the {MoC_4_N_2_} core of complex 2 are consistent with those observed in the handful of structures reported containing molybdenum tetracarbonyl complexes with chelating 2-(3-pyrazolyl)pyridine residues.^18^ The Mo^0^ centre is coordinated by four carbonyl groups and one *N*,*N*-chelating pyrazolylpyridine ligand, originating a distorted pseudo-octahedral geometry as confirmed by the distinct Mo–C and Mo–N bond lengths (Fig. 1e). The Mo–C bond lengths of the axial carbonyl groups (2.026(3), 2.042(3) Å) are longer than those for the equatorial groups (1.946(2), 1.954(2) Å), most probably as a consequence of distinct π back-bonding, Mo(d) → C≡O(π*). The equatorial CO groups are competing with the less π-acidic N-donor atoms from the pyrazolylpyridine ligand, while the axial groups compete with each other.^18*c*^ The geometric deformation of the octahedral coordination of the metal centre is further verified by an inspection of the internal bond angles: the *cis* angles are found between 71.71(6) and 101.82(8)°, while the *trans* angles are found in the range of 165.75(9)–173.41(8)°.

A full account of the supramolecular interactions (hydrogen bonding, π⋯π contacts, C–H⋯acceptor) and crystal packing arrangements in the crystal structures of [ptapzpy]Br, 1·CH_3_CN and 2·CH_3_CN is given in the ESI.†

### Oxidative decarbonylation

Oxidative decarbonylation of 1 was carried out by the dropwise addition of TBHP (10 equiv.) to a suspension of the complex in CH_2_Cl_2_. After stirring at room temperature for 4 h, an off-white solid (3) was recovered by filtration. Thermogravimetric analysis (TGA; Fig. S2 in the ESI†) and CHN microanalyses for 3 were consistent with the composition [Mo_3_O_9_([ptapzpy]Br)_2_]·3H_2_O. Powder XRD (PXRD) showed that the solid was amorphous, with only a few very broad overlapping diffraction peaks being observed in the 2*θ* range of 5–30° (Fig. S3†). The FT-IR and Raman spectra for 3 confirmed that complete decarbonylation had occurred during the reaction of 1 with the oxidant since no bands were observed in the carbonyl stretching region (1700–2020 cm^−1^; Fig. S4†). On the other hand, the spectra retained the characteristic ligand modes of ptapzpy in the range of 1000–1650 cm^−1^, matching closely the bands observed for 1 and therefore indicating that the ligand is coordinated in a bidentate fashion to Mo centres. The structural integrity of the organic ligand in 3 was verified by comparing its ^13^C{^1^H} CP MAS NMR spectrum with that of the free ligand [ptapzpy]Br (Fig. S5†). Treatment of complex 2 with TBHP under the same conditions as used for 1 gave rise to the same molybdenum oxide hybrid material 3 (in a comparable yield) on the basis of the similar TGA, PXRD and spectral data (Figures S2–S5†).

New vibrational bands for 3 that arise in the 850–1000 cm^−1^ interval are assigned to Mo–O vibrations. A very broad absorption band centred at 609 cm^−1^ in the IR spectrum is assigned to *ν*(Mo–O–Mo) and points towards a polynuclear or polymeric structure. Molybdenum oxide-organonitrogen hybrid materials having the general composition [Mo_3_O_9_(N–N)_2_] have been reported previously, namely the 1D materials with N–N = bipy^19*a*^ and 1,10-phenanthroline.^19*b*^ The Raman spectrum of [Mo_3_O_9_(bipy)_2_] was reported by Twu *et al.* and found to exhibit three Mo–O bands at 893, 923 and 946 cm^−1^ which coincide quite closely with the three bands observed for 3 at 895, 924 and 962 cm^−1^, suggesting that the two materials may possess the same type of molybdenum oxide substructure.^20^ The structure of the bipy hybrid consists of 1D chains built up from alternating {MoO_4_} tetrahedra and pairs of corner-linked {MoO_4_N_2_} octahedra.^19*a*^

### Catalytic epoxidation

**Scheme 2:**
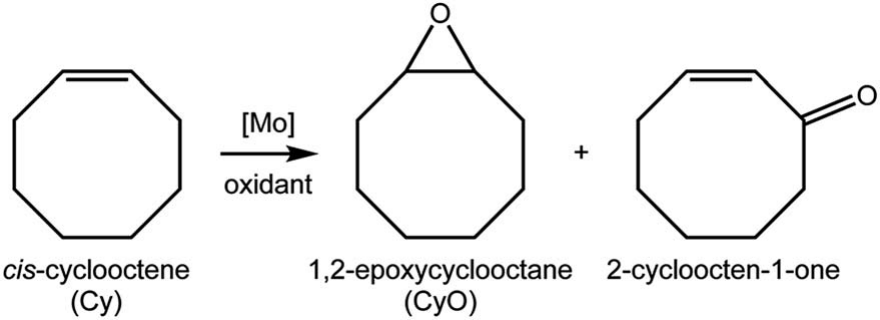
Reaction of *cis*-cyclooctene with TBHP, at 55 °C, in the presence of 1–3.

**Fig. 2:**
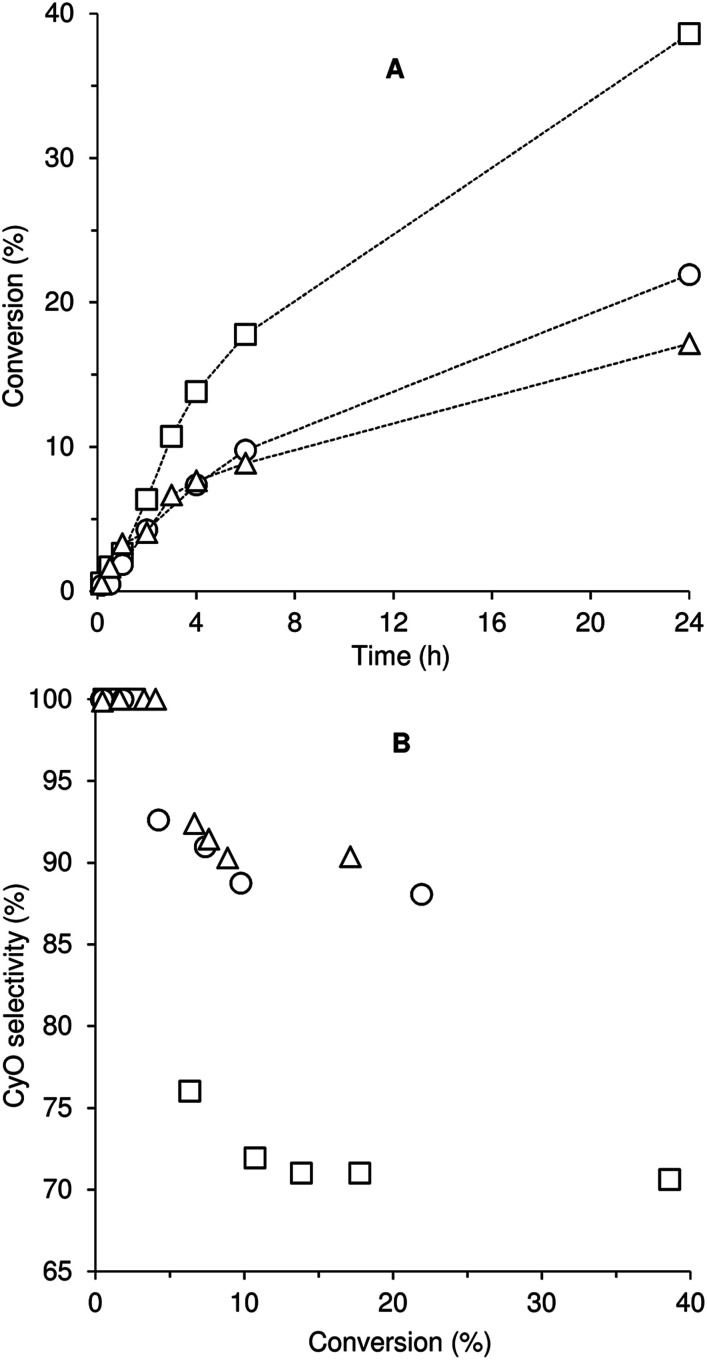
*cis*-Cyclooctene epoxidation with TBHP_D_ at 55 °C (DCE as cosolvent) using compounds 1 (□), 2 (○) and 3 (△): (A) conversion of Cy as a function of time and (B) selectivity of CyO as a function of Cy conversion. The dashed lines are visual guides.

Compounds 1–3 were tested for catalytic epoxidation with TBHP [decane solution (TBHP_D_) or aqueous solution (TBHP_A_)], at 55 °C, using *cis*-cyclooctene (Cy) as a benchmark substrate for olefins (Scheme 2). The three compounds led to 1,2-epoxycyclooctane (CyO) as the main product (Table 1). With 1,2-dichloroethane (DCE) as cosolvent and TBHP_D_ as oxidant, complex 1 led to a higher conversion at 24 h than 2 or 3 (39% compared with 22 and 17%, respectively). On the other hand, 1 led to lower CyO selectivity (71%) than 2 or 3 (86–88%), at 17–22% conversion (Fig. 2); byproducts included 2-cycloocten-1-one and others that were not clearly identified. Without catalyst, conversion was less than 10% at 24 h. The system TBHP_D_/DCE led to better results than TBHP_A_/CH_3_CN, in the presence of 1: 27% and 17% CyO yield, respectively, at 24 h. These contrasting performances may be partly due to differences in dissolution rate and solubility of the molybdenum compounds.

**Table 1:** *cis*-Cyclooctene epoxidation in the presence of 1–3^a^

Compound	Oxidant	Cosolvent	Conv.^b^ (%)	Yield^b^ (%)
—	TBHP_D_	None	-/-/4	-/-/4
1	TBHP_D_	DCE	3/18/39	3/13/27
1	TBHP_D_	None	9/35/54	9/31/47
1	TBHP_D_	[bmim]NTf_2_	6/25/46	6/22/40
1	TBHP_D_	[bmim]PF_6_	7/18/29	7/15/24
2	TBHP_D_	DCE	2/10/22	2/9/19
2	TBHP_D_	None	5/31/52	5/30/48
2^c^	TBHP_D_	None	-/31/48	-/28/45
2	TBHP_D_	[bmim]NTf_2_	4/16/37	4/16/35
3	TBHP_D_	DCE	3/9/17	3/8/15
3	TBHP_D_	None	5/23/40	5/22/38
3	TBHP_D_	[bmim]NTf_2_	3/13/34	3/13/31
3	TBHP_D_	[bmim]PF_6_	3/29/47	3/26/41
3^d^	TBHP_D_	[bmim]PF_6_	13/49/62	13/46/58
1	TBHP_A_	CH_3_CN	-/-/21	-/-/17
1	H_2_O_2_	CH_3_CN	11	9
1^d^	H_2_O_2_	CH_3_CN	20	16
2	H_2_O_2_	CH_3_CN	67	66
3	H_2_O_2_	CH_3_CN	56	56
3^d^	H_2_O_2_	CH_3_CN	51	51

a Reaction temperature = 55 °C and initial Mo : olefin : oxidant molar ratio = 1 : 113 : 172 (unless otherwise indicated), initial Cy concentration = 1.0 M (organic cosolvent and TBHP_A/D_), 1.7 M (ionic liquid and TBHP_D_), 1.2 M (CH_3_CN and H_2_O_2_) or 2.4 M (no cosolvent).

b Cy conversion and CyO yield at 1 h/6 h/24 h. Values for H_2_O_2_ as oxidant are for 24 h.

c Mo : olefin : oxidant molar ratio = 1 : 75 : 114.

d Reaction carried out at 70 °C.

The catalytic results were improved when no organic cosolvent was added: 87–95% CyO selectivity at 40–54% conversion, 24 h. Under these conditions, 1 and 2 display similar performance, leading to about 48% CyO yield at 24 h. For comparison, the complex *cis*-[Mo(CO)_4_(pzpy)] containing the unsubstituted organic ligand led to 78% epoxide yield under equivalent reaction conditions.^8*a*^ The catalytic results for 1 and 2 are intermediate between those reported in the literature (for similar reaction conditions: no cosolvent, 55 °C) for complexes of the type [Mo(CO)_*n*_(L)] containing chelating N-heterocyclic carbene (L) ligands (entries 6 and 7 of Table S3 in the ESI†), which led to 9–16% conversion at 24 h,^21^ and those for [Mo(CO)_4_(pyim)] (pyim = N-(*n*-propyl)-2-pyridylmethanimine), which led to 100% conversion at 5 h (entry 5 of Table S3†).^22^

The catalytic system 2/TBHP was further investigated for the epoxidation of styrene (which possesses a terminal C=C bond) and dl-limonene (which possesses endocyclic and exocyclic C=C bonds), at 55 °C, without additional solvent. Compound 2 led to 20%/39% styrene conversion at 6 h/24 h, and the main reaction products were styrene oxide and benzaldehyde formed with 52%/64% and 42%/31% selectivity, respectively. These results demonstrate the ability of 2/TBHP for the epoxidation of terminal C=C bonds, which are electron-richer.

The reaction of dl-limonene gave mainly limonene-1,2-oxide formed with 70%/71% selectivity at 23%/43% conversion, after 6 h/24 h, respectively. These results suggest regioselectivity effects in favour of the epoxidation of the endocyclic C=C bond over the exocyclic one. Other reaction products were formed *via* allylic oxidation, leading to alcohol and carbonyl derivatives of *p*-mentha-1,8-diene.

The use of the complex *cis*-[Mo(CO)_4_(pzpy)] in Cy epoxidation with TBHP_D_ led to a biphasic solid–liquid mixture and the solid phase was identified as the tetranuclear species [Mo_4_O_12_(pzpy)_4_].^8*a*^ Biphasic solid–liquid mixtures were also obtained for the systems (1, 2 or 3)/TBHP_D_. Attempts to isolate metal species from the colourless liquid phases were unsuccessful, suggesting that the concentration of dissolved species after a 24 h batch run was either negligible or very low. The low solubility of the metal species is probably one reason for the poor catalytic results obtained with these systems. Accordingly, for the system 2/TBHP_D_ (no cosolvent), a 1.5-fold increase in the catalyst amount (*i.e.* from *ca.* 0.9 mol% to 1.3 mol%) did not improve the epoxide yield (Table 1). This is because the catalytic reaction is homogeneous in nature and, for both catalyst amounts (which lead to biphasic solid–liquid reaction mixtures), the reaction mixtures are saturated with metal species.

**Fig. 3:**
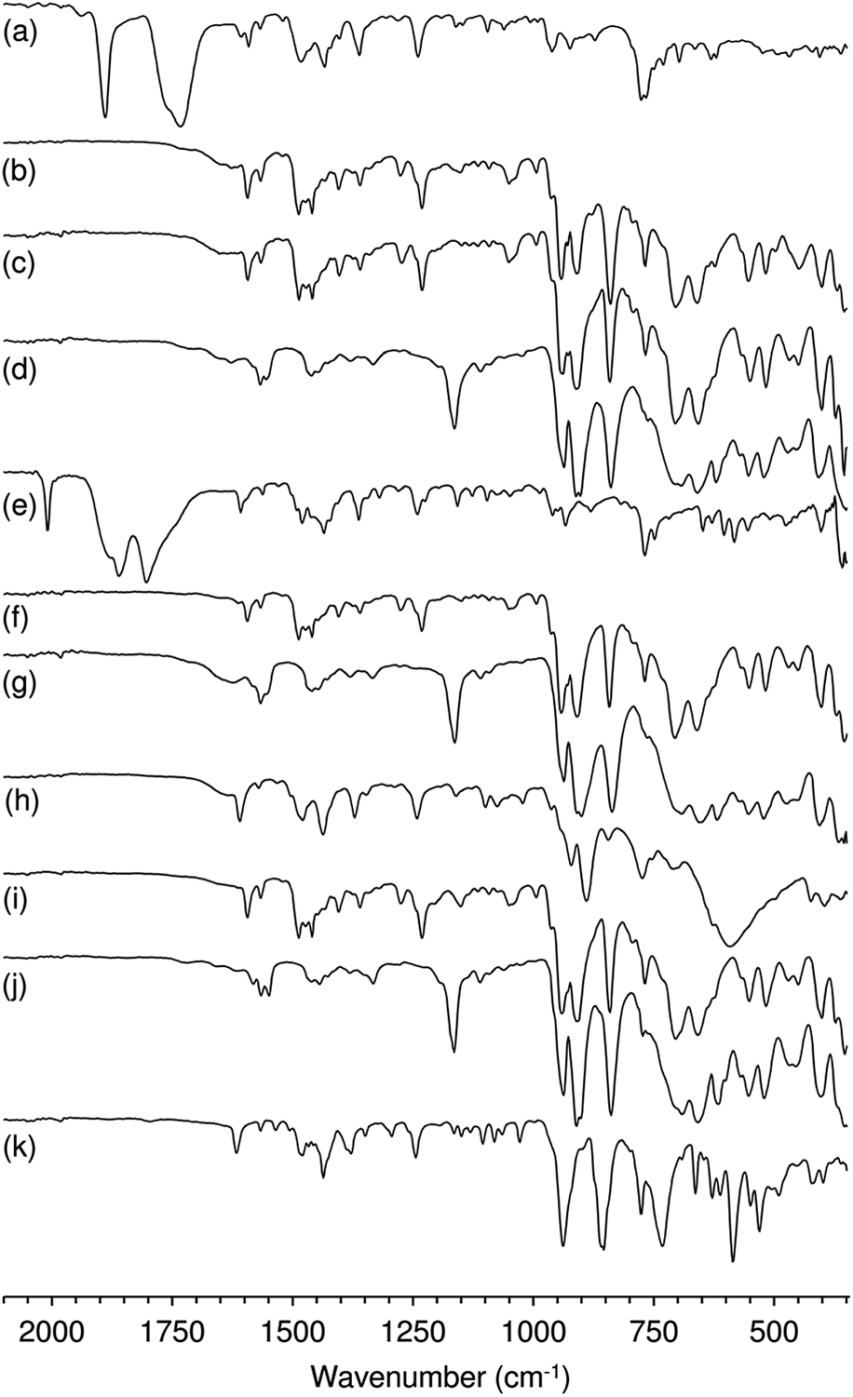
ATR FT-IR spectra of (a) 1, (e) 2 and (h) 3, and the recovered solids (b) 1-S-TBHP_D_, (c) 1-S-H_2_O_2_, (d) 1-S-TBHP_D_-[bmim]NTf_2_, (f) 2-S-TBHP_D_, (g) 2-S-TBHP_D_-[bmim]NTf_2_, (i) 3-S-TBHP_D_, (j) 3-S-TBHP_D_-[bmim]NTf_2_, and (k) 3-L-H_2_O_2_.

Characterisation of the off-white solid phases *i*-S-TBHP_D_ (*i* = 1–3; cosolvent = DCE) by ATR FT-IR spectroscopy indicated that 1–3 were converted to different types of metal species (Fig. 3). Comparison of these spectra with literature data for polyoxomolybdates of the type (L)_*x*_[Mo_8_O_26_] (where L is an organic cation such as pyridinium and imidazolium) indicates that a similar type of poorly soluble β-octamolybdate salt is formed in the catalytic reaction systems of 1–3 with TBHP_D_. In the region of the Mo–O vibrations (<1000 cm^−1^), the solids exhibited bands at *ca.* 942, 910, 840, 705 and 659 cm^−1^, which match closely with literature values for several (L)_*x*_[Mo_8_O_26_] salts (Table S4 in the ESI†).^23^ The recovered solids also exhibited a band at 767 cm^−1^ and several bands between 950 and 1650 cm^−1^ attributed to the charge-balancing organic cation ptapzpy. CHN microanalyses (Table S5 in the ESI†) for the solid 2-S-TBHP_D_ were consistent with the formulation (ptapzpy)_4_[Mo_8_O_26_]. PXRD data for this solid, designated as compound 4 in Scheme 1, showed that the material is microcrystalline (Fig. S6 in the ESI†).

Whereas an octamolybdate salt was formed from 1–3 under the catalytic reaction conditions with TBHP_D_, the oxidative decarbonylation of 1 and 2 with TBHP_D_ in the absence of substrate gave 3. These contrasting outcomes may be attributed to the different reaction conditions used (10 eq. TBHP_D_, room temperature/4 h and no substrate for the preparation of 3; 172 eq. TBHP_D_, 55 °C/24 h and presence of Cy for the catalytic reaction). It is still possible that in the catalytic reaction the hybrid material 3 may be an intermediate in the conversion of 1 and 2 to species of the type (L)_*x*_[Mo_8_O_26_].

Compounds 1–3 were further tested for Cy epoxidation with TBHP_D_ using the ionic liquid (IL) [bmim]NTf_2_ (bmim = 1-butyl-3-methylimidazolium; NTf_2_ = bis(trifluoromethylsulfonyl)imide) as cosolvent at 55 °C (Table 1). The use of an IL may enhance the solubility of the metal species, and thus the overall reaction kinetics. In comparison to the volatile organic solvent DCE, the reaction using [bmim]NTf_2_ as cosolvent led to superior catalytic results for all three compounds (Table 1). CyO selectivity of at least 87% was reached at 34–46% conversion (31–40% CyO yield). Complex 1 with [bmim]NTf_2_ led to superior catalytic results than those with [bmim]PF_6_, whereas the opposite was observed for 3. These results may be due to differences in solubility and rate of formation of the molybdenum species in the different ILs, although this is not trivial to demonstrate since it would require tracking the molybdenum species formed over time, and assessing their intrinsic activities. For the best performing system 3/[bmim]PF_6_, increasing the reaction temperature from 55 to 70 °C led to an increase in the CyO yield at 24 h from 41% to 58%.

After 24 h-batch runs at 55 °C, the reaction mixtures were liquid (IL)-liquid (organic phase)-solid(S). The ATR FT-IR spectra of the recovered solids *i*-S-TBHP_D_-[bmim]NTf_2_ were similar to the spectra of *i*-S-TBHP_D_ (*i* = 1–3) with respect to Mo–O vibrations (700–1000 cm^−1^), but different in the region of the ligand modes (>1000 cm^−1^) (Fig. 3). Similar results were obtained for *i*-S-TBHP_D_-[bmim]PF_6_ (not shown). The spectra very closely match that reported previously for the solid obtained upon OD of the tetracarbonyl complex *cis*-[Mo(CO)_4_(ppzpy)] (ppzpy = 2-(1-pentyl-3-pyrazolyl)pyridine) during Cy epoxidation with TBHP_D_ in the presence of [bmim]NTf_2_.^8*b*^ CHN microanalyses (Table S5 in the ESI†) for the solid 2-S-TBHP_D_-[bmim]NTf_2_ were consistent with the formulation (bmim)_3_(H_3_O)[Mo_8_O_26_]. PXRD data for this solid, designated as compound 5 in Scheme 1, showed that the material is microcrystalline (Fig. S6 in the ESI†). A comparison of the PXRD pattern of 5 with a simulated pattern calculated using crystal structure data reported for (bmim)_3_NH_4_[Mo_8_O_26_] (6) suggests that the two salts may have similar structures.^24^ Accordingly, FT-IR spectra for the two compounds display a striking correspondence in the region 350–1000 cm^−1^ (Fig. S7 in the ESI†). For 1–3 (Scheme 1), the precipitation of the β-octamolybdate salt with [bmim]^+^ counterions (rather than a cation derived from the ligand ptapzpy) is due to the large excess of IL present in the reaction mixture.

Compounds 1–3 were further studied for Cy reaction with H_2_O_2_ at 55 °C. Results with 2 and 3 (56–66% CyO yield at 24 h) were superior to those with 1 (9%) (Table 1). With the exception of complex 1, the catalytic results are generally better with H_2_O_2_ as oxidant than with TBHP. Notably, the CyO yield of 66% obtained with 2 surpasses the 41% yield obtained with *cis*-[Mo(CO)_4_(ppzpy)] under identical reaction conditions.^8*b*^

Iodometric titrations performed for 3/TBHP_D_/55 °C and 3/H_2_O_2_/55 °C (without substrate) showed that the decomposition of TBHP was not considerable (31% TBHP conversion at 24 h), whereas that of H_2_O_2_ was very significant (*ca.* 98% decomposition). Hence, the epoxidation process with TBHP does not seem to be compromised by side-reactions of the oxidant (*i.e.* unproductive decomposition into molecular oxygen and *tert*-butanol). On the other hand, the unproductive decomposition of H_2_O_2_ (into molecular oxygen and H_2_O) may compete with use of the oxidant for the catalytic reaction. Increasing the reaction temperature from 55 to 70 °C for the systems (1 or 3)/H_2_O_2_ was only slightly beneficial for 1 and actually led to poorer results with 3 (Table 1), presumably due to enhanced decomposition of the oxidant.

The differences in catalytic results for TBHP or H_2_O_2_ as oxidant may also be partly due to differences in catalyst stability. The system 1/H_2_O_2_ consisted initially of a yellow solution which changed with time into a biphasic solid–liquid mixture, where the liquid phase was colourless (attempts to isolate metal species from this phase were unsuccessful) and the solid was off-white. The FT-IR spectrum of the recovered solid 1-S-H_2_O_2_ (at 24 h) indicated that it was a salt of the type (L)_*x*_[Mo_8_O_26_] (similar to that verified for the systems (1–3)/TBHP) (Fig. 3c). For 2/H_2_O_2_ and 3/H_2_O_2_ the reaction mixture consisted of yellow liquid phases and no undissolved solid. The conversion of 2 and 3 to a different, highly soluble Mo-containing species may explain the superior catalytic results obtained for these systems. For 3/H_2_O_2_, a yellow solid was isolated from the liquid phase (3-L-H_2_O_2_), which exhibited a different ATR FT-IR spectrum from those of 3 and the recovered solids discussed above (Fig. 3k). The yellow colour is typically indicative of oxoperoxomolybdenum(vi) species. Accordingly, bands in the IR spectrum of the recovered solid may be assigned as *ν*(Mo=O) at 938 cm^−1^, *ν*(O–O) at 855 cm^−1^, and *ν*(Mo(O_2_)_2_) at 663, 585 and 530 cm^−1^. An additional strong and broad band at 732 cm^−1^ may be due to a Mo–O–Mo stretching vibration, suggesting the presence of a polynuclear species. From the characterisation studies, it seems that 1 possesses a different reactivity with H_2_O_2_ than 3, being converted to different types of metal species.

## Conclusions

Two distinct synthetic pathways (both starting with Mo(CO)_6_) have been established for the synthesis of molybdenum(0) tricarbonyl and tetracarbonyl complexes containing a bidentate-coordinated pyrazolylpyridine ligand. The complexes display moderate activity when applied as (pre)catalysts for the epoxidation of *cis*-cyclooctene with TBHP, which can be attributed to the *in situ* formation of poorly soluble β-octamolybdate salts. Better results were obtained with H_2_O_2_ as oxidant and the tetracarbonyl precatalyst, despite significant non-productive decomposition of the oxidant. The active species are yellow oxoperoxo-molybdenum(vi) species formed by the oxidative decarbonylation of the precursor. Treatment of the carbonyl precursors with TBHP in the absence of olefin gave a molybdenum oxide–organonitrogen hybrid material with a substructure that is proposed to consist of {MoO_4_} tetrahedra and {MoO_4_N_2_} octahedra. Work is ongoing to isolate a more crystalline form of this hybrid and determine its structure.

## Experimental

### Synthesis

#### 2-(1-Propyltrimethylammonium-3-pyrazolyl)pyridine bromide ([ptapzpy]Br)

2-[3(5)-Pyrazolyl]pyridine (2.00 g, 13.78 mmol) was added slowly to a suspension of NaH (0.5 g, 20.8 mmol) in THF (40 mL), resulting in a yellow mixture. A solution of (3-bromopropyl)trimethylammonium bromide (3.60 g, 13.79 mmol) in acetonitrile (90 mL) was added dropwise and the mixture stirred under reflux for 4 h, resulting in a yellow solution and a white precipitate. The mixture was evaporated to dryness under reduced pressure. The residue was extracted with CHCl_3_ (4 × 50 mL) and the combined extracts were evaporated to dryness under reduced pressure, giving a brown oil. The oil was washed with diethyl ether (50 mL) and then dissolved in acetone (50 mL). Diethyl ether (50 mL) was added to precipitate the product, which was filtered, washed with diethyl ether/acetone, and finally vacuum-dried to give the ligand [ptapzpy]Br as a cream solid. Yield: 2.0 g, 45%. Anal. calcd for C_14_H_21_BrN_4_·1.6H_2_O (354.07): C, 47.49; H, 6.89; N, 15.82. Found: C, 47.60; H, 6.45; N, 15.74%. FT-IR (KBr, cm^−1^): 404 (m), 470 (w), 524 (w), 619 (m), 630 (m), 698 (m), 728 (m), 750 (w), 777 (vs), 871 (m), 923 (m), 962 (s), 970 (s), 991 (w), 1037 (w), 1060 (m), 1095 (m), 1147 (w), 1160 (m), 1189 (w), 1240 (s), 1282 (w), 1303 (w), 1330 (w), 1359 (s), 1403 (m), 1432 (s), 1450 (w), 1465 (w), 1490 (s), 1519 (m), 1567 (m), 1590 (s) (*ν*_C=N_), 1649 (w), 2954 (m), 3002 (m), 3024 (m), 3083 (m), 3118 (w). ^1^H NMR (300.1 MHz, 295 K, CDCl_3_): *δ* = 8.63 (d, 1H, H-11), 7.83 (td, 1H, H-8), 7.73 (dt, 1H, H-9), 7.64 (d, 1H, H-5), 7.24 (ddd, 1H, H-10), 6.84 (d, 1H, H-4), 4.41 (t, 2H, N–CH_2_), 3.80 (m, 2H, N–CH_2_), 3.39 (s, 9H, N–CH_3_), 2.55 (m, 2H, CH_2_) ppm (see Fig. S5 in the ESI† for atom numbering). ^13^C NMR (75.4 MHz, 295 K, CDCl_3_): *δ* = 152.51 (C-7), 152.07 (C-11), 149.76 (C-3), 136.92 (C-9), 132.32 (C-5), 122.82 (C-8), 120.37 (C-10), 104.91 (C-4), 64.48 (N–CH_2_), 53.88 (N–CH_3_), 48.73 (N–CH_2_), 24.26 (CH_2_) ppm. ^13^C{^1^H} CP MAS NMR: *δ* = 151.3 (C-7), 149.6 (C-11 and C-3), 138.0 (C-9), 129.3 (C-5), 124.4 (C-8), 121.1 (C-10), 107.4 (C-4), 61.2 (N–CH_2_), 53.7 (N–CH_3_), 47.8 (N–CH_2_), 24.7 (CH_2_) ppm.

#### [Mo(CO)_3_(ptapzpy)Br] (1)

In a Schlenk tube, Mo(CO)_6_ (0.20 g, 0.76 mmol) and [ptapzpy]Br (0.25 g, 0.77 mmol) were added to toluene (20 mL) and the mixture was refluxed under N_2_ for 30 min, resulting in an orange solid and solution. Hexane (20 mL) was added to promote product precipitation. The solution was filtered off and the solid washed with hexane (2 × 20 mL), diethyl ether (2 × 20 mL), and finally vacuum-dried. Yield: 0.34 g, 83%. Anal. calcd for C_17_H_21_BrMoN_4_O_3_·2H_2_O (541.25): C, 37.72; H, 4.66; N, 10.35. Found: C, 37.58; H, 4.81; N, 10.1%. TGA (Fig. S2†) showed a mass loss of 6.2% at 100 °C (calcd for loss of 2H_2_O: 6.6%). Selected FT-IR (KBr, cm^−1^): 494 (w), 629 (w), 771 (m), 873 (w), 960 (m), 1095 (w), 1157 (w), 1239 (m), 1365 (m), 1439 (m), 1477 (m), 1604 (w) (*ν*_C=N_), 1742 (vs) (*ν*_CO_), 1764 (vs) (*ν*_CO_), 1895 (vs) (*ν*_CO_).

#### [Mo(CO)_4_(ptapzpy)]Br (2)

[Mo(CO)_4_(pip)_2_] (0.11 g, 0.30 mmol) was added to a solution of [ptapzpy]Br (0.10 g, 0.30 mmol) in ethanol (10 mL) and the mixture was heated at 50 °C, with stirring, for 30 min. The solvents were evaporated under reduced pressure, and the resultant red solid was washed with hexane (2 × 7 mL) and ethanol (2 × 7 mL). Yield: 0.13 g, 79%. Anal. calcd for C_18_H_21_BrMoN_4_O_4_·1.5H_2_O (560.25): C, 38.59; H, 4.32; N, 10.00. Found: C, 38.90; H, 4.58; N, 10.14%. TGA (Fig. S2†) showed a mass loss of 4.2% at 90 °C (calcd for loss of 1.5H_2_O: 4.8%). Selected FT-IR (KBr, cm^−1^): 364 (s), 471 (w), 580 (m), 650 (m), 765 (s), 962 (m), 1097 (w), 1241 (m), 1365 (m), 1438 (m), 1482 (w), 1608 (w) (*ν*_C=N_), 1815 (vs) (*ν*_CO_), 1869 (vs) (*ν*_CO_), 1888 (sh) (*ν*_CO_), 2012 (s) (*ν*_CO_).

#### [Mo_3_O_9_([ptapzpy]Br)_2_] (3)

In a Schlenk tube, 5–6 M TBHP in decane (0.35 mL, *ca.* 2 mmol) was added to a mixture comprising complex 1 (0.10 g, 0.20 mmol) and CH_2_Cl_2_ (10 mL), and stirring was continued for 4 h under N_2_ at room temperature. The resultant off-white solid was recovered by filtration, washed with diethyl ether (2 × 10 mL), and finally vacuum-dried. Yield: 0.04 g, 53%. Anal. calcd for C_28_H_42_Br_2_Mo_3_N_8_O_9_·3H_2_O (1136.36): C, 29.59; H, 4.26; N, 9.86. Found: C, 29.51; H, 4.47; N, 9.99%. TGA (Fig. S2†) showed a mass loss of 5.5% at 150 °C (calcd for loss of 3H_2_O: 4.8%). Selected FT-IR (KBr, cm^−1^): 326 (w), 399 (w), 426 (w), 609 (s), 631 (s), 777 (m), 846 (w), 895 (s), 924 (s), 962 (w), 1022 (w), 1076 (w), 1099 (w), 1161 (w), 1242 (m), 1371 (m), 1439 (m), 1479 (m), 1610 (m) (*ν*_C=N_), 2850 (w), 3026 (w). Selected FT-Raman (cm^−1^): 641 (w), 727 (w), 752 (w), 783 (m), 893 (w), 923 (vs), 963 (m), 1020 (m), 1053 (w), 1159 (w), 1242 (w), 1287 (w), 1346 (m), 1372 (m), 1422 (m), 1532 (m), 1568 (m), 1609 (m) (*ν*_C=N_), 2826 (w), 2966 (m), 3022 (m), 3065 (m). ^13^C{^1^H} CP MAS NMR: *δ* = 146.8 (C-7, C-11 and C-3), 140.5 (C-9), 134.7 (C-5), 124.8 (C-8 and C-10), 105.2 (C-4), 63.8 (N–CH_2_), 53.6 (N–CH_3_), 48.9 (N–CH_2_), 25.2 (CH_2_) ppm.

### Catalytic tests

The typical epoxidation experiments with TBHP (in decane or aqueous solution) were carried out in 10 mL borosilicate batch reactors possessing a valve for sampling. Reaction mixtures were stirred magnetically (1000 rpm, PTFE-coated stirring bar) and heated to 55 °C with a thermostatically controlled oil bath. The reactors were charged with the (pre)catalyst (amount equivalent to 16 μmol Mo), *cis*-cyclooctene (1.8 mmol), and a cosolvent [1 mL of 1,2-dichloroethane or CH_3_CN, or 0.3 mL of an ionic liquid (IL)]. The reactor containing the catalyst, olefin and cosolvent was preheated for 10 min at the reaction temperature. In a separate flask, the oxidant was preheated in a similar fashion, and subsequently added to the reactor to give a Mo : Cy : oxidant molar ratio of 1 : 113 : 172; this was marked as the initial instant of the catalytic reaction.

Catalytic tests using 30 wt% aq. H_2_O_2_ as oxidant were carried out using tubular borosilicate batch reactors with pear-shaped bottoms (*ca.* 12 mL capacity), equipped with a PTFE-coated magnetic stirring bar (1000 rpm) and a valve for (un)charging of the reactor. Catalyst (16 μmol Mo), Cy (1.8 mmol), CH_3_CN (1 mL) and H_2_O_2_ (Mo : Cy : oxidant molar ratio of 1 : 113 : 172) were added to the reactor, which was subsequently immersed in an oil bath heated to 55 or 70 °C. Separate catalytic experiments were carried out for each reaction time.

The reaction mixtures were analysed by using a Varian 3900 GC equipped with a DB-5 capillary column (30 m × 0.25 mm × 0.25 μm) and a FID detector, with H_2_ as the carrier gas. The concentrations of reactant and products were determined using the internal calibration method, *i.e.* based on calibration curves with undecane as internal standard. The FID response was linear in the range of concentrations used for the calibration curves and sample analysis. Using the internal calibration method the determined concentrations of Cy and CyO are reliable (experimental range of error of *ca.* 6%), *i.e.* the conversion values account for substrate consumption irrespective of the types of products formed being detected or not by GC. The reactant/products were identified using GC-MS (Trace GC 2000 Series Thermo Quest CE Instruments GC; Thermo Scientific DSQ II), using He as the carrier gas.

Iodometric titrations were carried out in order to quantify the non-productive decomposition of the oxidants (TBHP or H_2_O_2_). The reactors containing the (pre)catalyst, solvent and oxidant, without substrate, were heated at 55 °C for 24 h. After cooling the reactors to ambient temperature, liquid samples were withdrawn for titration.

For reaction mixtures which were biphasic solid–liquid, the solid phase was separated from the catalytic reaction mixture by centrifugation (3500 rpm), washed with organic solvents (pentane, hexane, ethanol and/or acetone), dried overnight under atmospheric conditions, and subsequently under vacuum (*ca.* 4 mbar) for 1 h at 60 °C. The recovered solids are denoted *i*-S-oxid where oxid is the oxidant (TBHP or H_2_O_2_) and *i* is compound 1, 2 or 3. For epoxidation systems using ionic liquids, the recovered solids are denoted *i*-S-TBHP_D_-IL {IL = [bmim]NTf_2_ or [bmim]PF_6_}. For the system 3/H_2_O_2_, metal species could be isolated from the liquid phases of the catalytic reactions by precipitation of solids after addition of an appropriate organic solvent. The precipitated solid was washed and dried as described above for the recovery of the undissolved solids and denoted 3-L-H_2_O_2_.

## Conflicts of interest

There are no conflicts to declare.

## Supplementary Material

RA-008-C8RA01687A-s001

RA-008-C8RA01687A-s002
