# Supplementary material for: Molybdenum(0) tricarbonyl and tetracarbonyl complexes with a cationic pyrazolylpyridine ligand: synthesis, crystal structures and catalytic performance in olefin epoxidation
Source: RSC Adv. 2018 May 1;8(29):16294–302. doi: 10.1039/c8ra01687a (PMC9080237; doi:10.1039/c8ra01687a)
Supplement: RA-008-C8RA01687A-s001 [file RA-008-C8RA01687A-s001.pdf]

Electronic Supplementary Material (ESI) for RSC Advances.
This journal is © The Royal Society of Chemistry 2018

Electronic Supporting Information

Molybdenum(0) Tricarbonyl and Tetracarbonyl Complexes with a Cationic Pyrazolylpyridine Ligand: Synthesis, Crystal Structures and Catalytic Performance in Olefin Epoxidation

Lucie S. Nogueira,[a] Patrícia Neves,[a] Ana C. Gomes,[a] Pedro Lavrador,[a] Luís Cunha-Silva,[b] Anabela A. Valente,*[a] Isabel S. Gonçalves[a] and Martyn Pillinger*[a]

a Department of Chemistry, CICECO - Aveiro Institute of Materials, University of Aveiro, Campus Universitário de Santiago, 3810-193 Aveiro, Portugal
E-mail: atav@ua.pt (A.A.V.); mpillinger@ua.pt (M.P.)

b REQUIMTE/LAQV & Department of Chemistry and Biochemistry, Faculty of Sciences, University of Porto, 4169-007 Porto, Portugal

Contents

Pages S2	**Additional experimental details**<br>Chemicals and physical measurements.
Pages S2-S6	**Single-Crystal X-Ray Diffraction Studies**<br>Experimental, Crystal and structure refinement data (Table S1), Supramolecular interactions and crystal packing (Fig. S1 and Table S2).
Page S7	**Figure S2.** TGA curves of tricarbonyl complex **1**, tetracarbonyl complex **2**, and the hybrid material **3**.
Page S7	**Figure S3.** Powder X-ray diffraction patterns of the hybrid material **3**.
Page S8	**Figure S4.** FT-IR and FT-Raman spectra of [ptapzpy]Br, tricarbonyl complex **1**, tetracarbonyl complex **2**, and the hybrid material **3**.
Page S9	**Figure S5.** ^{13}C solution and $^{13}C\{^1H\}$ CP MAS NMR spectra.
Page S10	**Table S3.** Catalytic performance of compounds **1** and **2** compared with selected literature data.
Page S11	**Table S4.** Comparison of FT-IR bands observed for recovered solids with corresponding bands for compounds containing the β-$[Mo_8O_{26}]^{4-}$ anion.
Page S11	**Table S5.** Elemental analysis results.
Page S12	**Fig. S6 and Fig. S7.** Powder XRD and ATR FT-IR spectra of recovered solids and $(bmim)_3NH_4[Mo_8O_{26}]$.
Page S13	**References**

Additional experimental details

Chemicals: Hydrazine ($N_2H_4 \cdot H_2O$, hydrate solution, 80 %), NaH (60 % dispersion in mineral oil), piperidine (99.5 %, Carlo-Erba), (3-bromopropyl)trimethylammonium bromide (97 %), [bmim]NTf$_2$ (99 %, Iolitec), [bmim]PF$_6$ (99 %, Iolitec), Mo(CO)$_6$, 30 wt.% H_2O_2, 5-6 M TBHP in decane, 70 wt.% aq. TBHP, *cis*-cyclooctene (95 %, Alfa Aesar), styrene (≥99 %) and DL-limonene (≥95 %, Merck) were acquired from Sigma-Aldrich unless otherwise indicated, and used as received. Solvents were purchased from commercial suppliers and used as received (≥99 %, Sigma-Aldrich, Carlo-Erba and Riedl-de-Haën). 2-[3(5)-Pyrazolyl]pyridine[1] and *cis*-[Mo(CO)$_4$(pip)$_2$][2] were prepared as described previously. A literature procedure[3] for the preparation of (bmim)$_4$[Mo$_8$O$_{26}$] (involving addition of [bmim]Cl to an acidified solution of ammonium heptamolybdate) was followed but gave the compound (bmim)$_3$NH$_4$[Mo$_8$O$_{26}$] (**6**), which was recently reported by Linguito *et al.*[4]

Physical measurements: Microanalyses for C, H and N were carried out at the Department of Chemistry, University of Aveiro, with a Truspec Micro CHNS 630-200-200 elemental analyser. PXRD data were collected at ambient temperature using a PANalytical Empyrean instrument equipped with a PIXcel 1D detector set at 240 mm from the sample. Cu-K$_{\alpha1,2}$ X-radiation (λ_1 = 1.540598 Å; λ_2 = 1.544426 Å) filtered with a nickel foil was used along with a standard transmission sample holder. Working operating conditions for the X-ray tube: 45 kV and 40 mA. Intensity data were collected in continuous mode in the ca. $3.5 \leq 2\theta \leq 70°$ range. TGA was performed using a Shimadzu TGA-50 system at a heating rate of 5 °C min^{-1} under air. FT-IR spectra were recorded on a Mattson 7000 FT-IR spectrometer using KBr pellets. Attenuated total reflectance (ATR) FT-IR spectra were measured using a Specac Golden Gate Mk II ATR accessory having a diamond top plate and KRS-5 focusing lenses. FT-Raman spectra were recorded on an RFS-100 Bruker FT-Spectrometer equipped with a Nd:YAG laser with an excitation wavelength of 1064 nm. ^{1}H and ^{13}C solution NMR spectra were recorded on Bruker Avance 300 and 400 spectrometers. Chemical shifts are quoted in ppm from TMS. Solid-state $^{13}C\{^1H\}$ CP MAS NMR spectra were recorded using a Bruker Avance 400 (narrow bore) spectrometer with an ultrashielded static magnetic field of 100.6 MHz. The spectra were recorded with 2.75 µs ^{1}H 90° pulses, 2.0 ms contact time, spinning rate of 14 kHz, and 5 s recycle delays. Chemical shifts are quoted in ppm from TMS.

Single-Crystal X-Ray Diffraction Studies

Experimental: Single crystals of [ptapzpy]Br suitable for X-ray diffraction were obtained by slow diffusion of diethyl ether into a concentrated solution of [ptapzpy]Br in dichloromethane. To obtain single crystals of **1** and **2**, a mixture of Mo(CO)$_6$ (0.08 g, 0.30 mmol), [ptapzpy]Br (0.10 g, 0.30 mmol) and CH_3CN (10 mL) was refluxed under N_2 for 30 min,

and the resultant solution was filtered off. Yellow (**1**·CH_3CN) and orange (**2**·CH_3CN) single crystals were obtained after vapor diffusion of diethyl ether into the solution at ambient temperature.

Crystalline material of the compounds [ptapzpy]Br, [Mo(CO)$_3$(ptapzpy)Br]·CH_3CN (**1**·CH_3CN) and [Mo(CO)$_4$(ptapzpy)]Br·CH_3CN (**2**·CH_3CN) was harvested from the crystallization vials, immersed in Fomblin® Y oil, and a single-crystal of each sample was mounted on the respective CryoLoops with the support of a stereomicroscope.[5] Full diffraction data sets were obtained at 150(2) K on a Bruker X8 APEX II CCD area-detector diffractometer (Mo K_α graphite-monochromated radiation, λ = 0.71073 Å), with the acquisition controlled by APEX2 software.[6] Collected images were processed using SAINT+,[7] and absorption effects were corrected through the multi-scan semi-empirical method implemented in SADABS.[8] The crystal structures were solved using the algorithms implemented in SHELXT-2014,[9,10] and all non-H atoms were located from difference Fourier maps calculated from successive full-matrix least squares refinement cycles on F^2 using SHELXL-v.2014 (Mo, C, Br, N and O atoms were successfully refined with anisotropic displacement parameters).[9,11] The relatively long bond length of 2.072(9) Å for Mo-C17 (axial CO) in the structure of **1**·CH_3CN is due to a slight crystalline disorder between Br1 and C17-O3, which was not possible to modulate in the crystal refinement. The positional disorder of these two groups originates an increase in the Mo-C17 bond length. H-atoms connected to the distinct carbons were fixed at geometrical positions using the following *HFIX* instructions in SHELXL-v.2014: *137* for the terminal –CH_3, *23* for the –CH_2– and *43* for the aromatic groups, and included in refinement cycles in riding-motion approximation with isotropic thermal displacement parameters (U_{iso}) fixed at 1.2 $\times$ or 1.5 $\times$ U_{eq} of the parent C-atom. Table S1 shows selected information about the single-crystal X-ray data collection and the structure refinements.

Supramolecular interactions and crystal packing: The absence of hydrogen atoms attached to either oxygen or nitrogen in the ligand ptapzpy and the complexes **1** and **2** prevents the occurrence of strong hydrogen bonds in the three structures. Consequently the packing arrangement of the organic ligand (in the structure of [ptapzpy]Br) and complexes (in the structures of **1**·MeCN and **2**·MeCN) is basically driven by effective space filling closely mediated by an extensive network weak of C–H···Acceptor and π···π intermolecular interactions (Fig. S1; geometric details about weak hydrogen bonds are summarized in Table S2). The bromide anion in the three crystal structures, acting as either a counterion or metal-coordinated ligand, plays a decisive role in the establishment of a large number of such interactions.

In the extended structure of [ptapzpy]Br the adjacent ligands interact by weak C–H···N hydrogen bonds (light blue dashed lines in Fig. S1a). These intermolecular interactions lead to the formation of one-dimensional (1D) supramolecular networks (infinite chains) along the [0 1 0] direction of the unit cell (not shown). Furthermore, the bromide anions interconnect

these organic chains by numerous and distinct C–H··Br interactions (drawn as light green dashed lines) ultimately leading to a 3D supramolecular network.

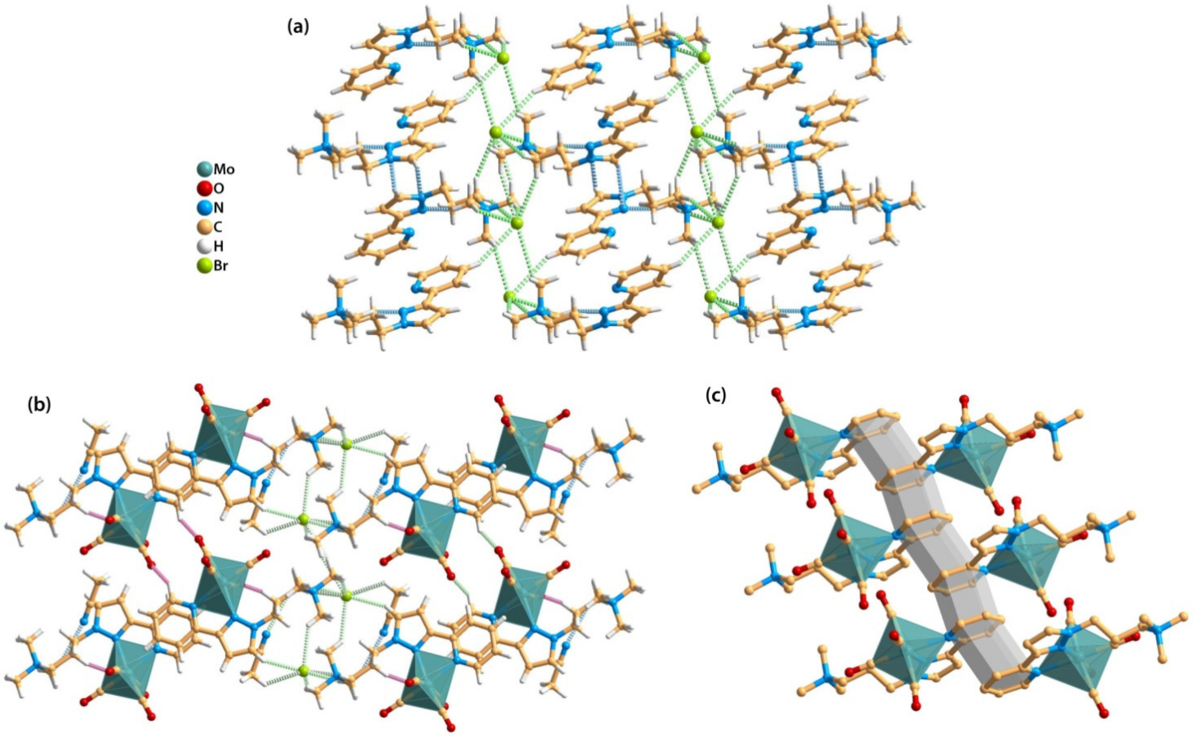

Fig. S1 Representation of selected features of the extended crystal structures: (a) C–H···N and C–H···Br hydrogen bonds in [ptapzpy]Br; (b) C–H···O, C–H···N and C–H···Br interactions in **2**·CH_3CN; (c) π···π stacking between the pyridine rings of adjacent complexes **2** (grey shadow). Weak C–H···O, C–H···N and C–H···Br hydrogen bonds are shown as pink, blue and green dashed lines, respectively. The geometric details of these interactions are listed in Table S2.

The packing arrangement of neighbouring cationic complexes $[Mo(CO)_4$(2-(1-propyltrimethylammonium-3-pyrazolyl)pyridine)$]^+$ in **2**·CH_3CN is driven by C–H···O intermolecular interactions (light pink dashed lines in Fig. S1b) involving the carbonyl groups, as well as by π···π stacking between the pyridine rings of adjacent complexes [*C*g···*C*g distances of 3.7901(6) and 3.8623(6) Å; grey shadow in Fig. S1c]. Furthermore, the CH_3CN molecule of crystallisation establishes C–H··N interactions with the complexes (light blue dashed lines in Fig. S1b). In a mode analogous to that described for [ptapzpy]Br, the bromide anions are involved in an extensive network of weak C–H··Br hydrogen bonds (light green dashed lines in Fig. S1b), strengthening the cohesion of the 3D supramolecular structure of the compound **2**·CH_3CN. Similarly, the packing arrangement of adjacent neutral complexes $[Mo(CO)_3$(ptapzpy)Br] (**1**) is also driven by diverse C–H···O, C–H···O and C–H···Br weak hydrogen bonds, involving adjacent complexes and also the CH_3CN molecules (not shown; for geometric details about these interactions see Table S2).

Table S1 Crystal and structure refinement data for the compounds [ptapzpy]Br, [Mo(CO)$_3$(ptapzpy)Br]·CH_3CN (**1**·CH_3CN) and [Mo(CO)$_4$(ptapzpy)]Br·CH_3CN (**2**·CH_3CN).

	[ptapzpy]Br	**1**·CH_3CN	**2**·CH_3CN
Chemical formula	$C_{14}H_{21}BrN_4$	$C_{19}H_{24}BrMoN_5O_3$	$C_{20}H_{24}BrMoN_5O_4$
M_r	325.26	546.28	574.23
Crystal description	Colorless prism	Yellow prism	Orange prism
Crystal size/mm	0.19 × 0.07 × 0.05	0.08 × 0.02 × 0.02	0.08 × 0.05 × 0.03
Crystal system, space group	Monoclinic, $P2_1/c$	Monoclinic, $P2_1$	Triclinic, P−1
a/Å	9.0727(4)	7.4060(6)	7.5420(12)
b/Å	7.6409(3)	9.7868(7)	8.9322(12)
c/Å	22.0574(10)	15.3808(8)	17.705(3)
α/°	90	90	87.812(7)
β/°	94.711(2)	97.385(4)	88.701(8)
γ/°	90	90	87.216(7)
Volume/Å^3	1523.93(11)	1105.6(2)	1190.2(3)
Z	4	2	2
$\rho_{calculated}$/g cm^{-3}	1.418	1.641	1.602
F(000)	672	548	576
μ/mm^{-1}	2.691	2.429	2.264
θ range/°	3.659 to 30.033	3.843 to 28.278	3.688 to 26.370
Index ranges	$-12 \le h \le 12$	$-9 \le h \le 8$	$-9 \le h \le 9$
	$-10 \le k \le 10$	$-13 \le k \le 11$	$-11 \le k \le 11$
	$-30 \le l \le 31$	$-20 \le l \le 20$	$-22 \le l \le 22$
Reflections collected	55072	18898	40010
Independent reflections	4440	5075	4843
	(R_{int} = 0.0311)	(R_{int} = 0.0477)	(R_{int} = 0.0364)
Final R indices [I>2σ(I)]	R_1 = 0.0232	R_1 = 0.0412	R_1 = 0.0241
	wR_2 = 0.0511	wR_2 = 0.0825	wR_2 = 0.0529
Final R indices (all data)	R_1 = 0.0386	R_1 = 0.0578	R_1 = 0.0313
	wR_2 = 0.0564	wR_2 = 0.0889	wR_2 = 0.0555
$\Delta\rho_{max}$ and $\Delta\rho_{min}$/e Å^{-3}	0.363 and −0.212	1.313 and −0.701	0.776 and −0.550

Table S2 Geometric information for the C–H···A hydrogen bonding interactions of the compounds [ptapzpy]Br, **1**·CH_3CN and **2**·CH_3CN.[a]

Compound	C–H···A	*d*(H···A)/Å	*d*(C···A)/Å	∠(CHA)/°
[ptapzpy]Br	C4–H4···$Br1^{i}$	2.97	3.9123(14)	172.0
	C7–H7···$Br1^{i}$	2.87	3.8173(14)	172.9
	C8–H8···$N2^{ii}$	2.68	3.4852(19)	143.2
	C11–H11A···Br1	2.73	3.6717(13)	159.5
	C11–H11B···$Br1^{iii}$	3.00	3.9100(13)	153.0
	C12–H12A···$Br1^{iv}$	3.08	3.5585(15)	111.8
	C13–H13A···$Br1^{v}$	2.93	3.8404(15)	154.6
	C13–H13B···$N2^{v}$	2.58	3.2034(18)	121.3
	C14–H14A···$Br1^{iii}$	2.95	3.8783(16)	159.3
	C14–H14C···$Br1^{v}$	2.88	3.8003(14)	156.9
**1**·CH_3CN	C8–H8···$N5^{vi}$	2.53	3.434(10)	158.4
	C9–H9A···$Br1^{vii}$	3.09	3.573(6)	111.5
	C10–H10A···Br1	2.97	3.940(6)	165.1
	C10–H10B··$Br1^{vii}$	3.03	3.650(6)	121.9
	C12–H12B···$O2^{ix}$	2.57	3.509(11)	160.7
	C12–H12B···$Br1^{viii}$	3.11	3.987(9)	149.4
	C13–H13A···$Br1^{viii}$	2.98	3.849(8)	148.7
	C13–H13C···$O2^{viii}$	2.62	3.443(10)	141.8
	C14–H14C···$Br1^{viii}$	2.98	3.881(7)	154.0
	C19–H19A···$Br1^{x}$	2.84	3.780(10)	160.2
	C19–H19B···$O2^{xi}$	2.47	3.441(10)	169.4
	C19–H19C···$O3^{xi}$	2.59	3.348(10)	134.5
**2**·CH_3CN	C1–H1···$O3^{xii}$	2.54	3.272(3)	134.1
	C8–H8···$Br1^{xiii}$	2.88	3.736(2)	150.5
	C10–H10A···$O4^{xiv}$	2.53	3.266(3)	130.8
	C11–H11B···$Br1^{xv}$	2.83	3.773(3)	159.6
	C12–H12A···$Br1^{xiii}$	2.90	3.589(3)	128.0
	C13–H13A···$Br1^{xv}$	2.94	3.842(3)	154.2
	C13–H13B···Br1	2.79	3.751(2)	166.2
	C14–H14A···$Br1^{xvi}$	2.87	3.826(3)	165.7
	C14–H14C···$N5^{xiii}$	2.65	3.582(4)	158.6
	C19–H19A···Br1	2.97	3.592(3)	122.3

[a] Symmetry transformations used to generate equivalent atoms: (i) *x, −y+3/2, z*−1/2*;* (ii) *−x+1, y+1/2, −z+1/2;* (iii) −x+1, −y+2, −z+1; (iv) *−x, −y+2, −z+1*; (v) *x, y+1, z*; (vi) *−x+1, y−1/2, −z+1;* (vii) *x−1, y, z;* (viii) *−x+1, y−1/2, −z+2;* (ix) *x−1, y−1, z;* (x) *−x+2, y+1/2, −z+1;* (xi) *−x+2, y−1/2, −z+1;* (xii) *−x+2, −y+1, −z;* (xiii) *x, y+1, z*; (xiv) *x−1, y, z;* (xv) *−x+1, −y+1, −z+1;* (xvi) *−x, −y+1, −z+1.*

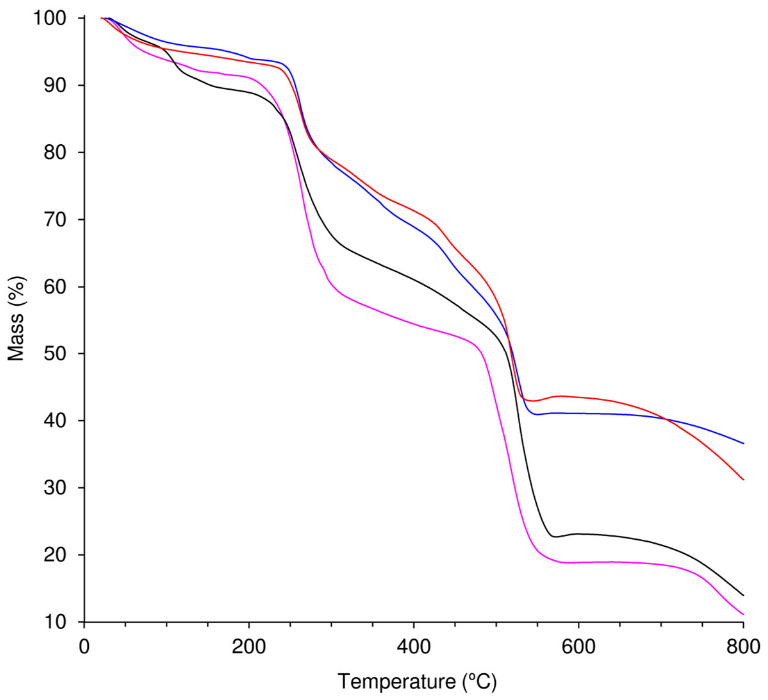

Fig. S2 TGA curves of the tricarbonyl complex **1** (▬▬), tetracarbonyl complex **2** (▬▬), and the hybrid material **3** prepared from **1** (▬▬) and **2** (▬▬).

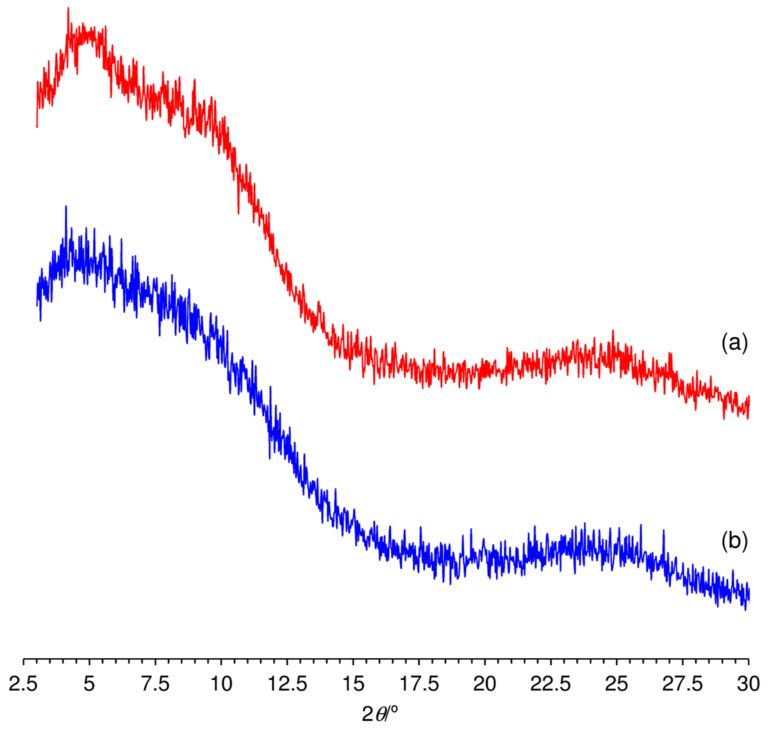

Fig. S3 Powder X-ray diffraction patterns in the range of 2.5-30° 2θ of the hybrid material **3** prepared from **1** (▬▬) and **2** (▬▬).

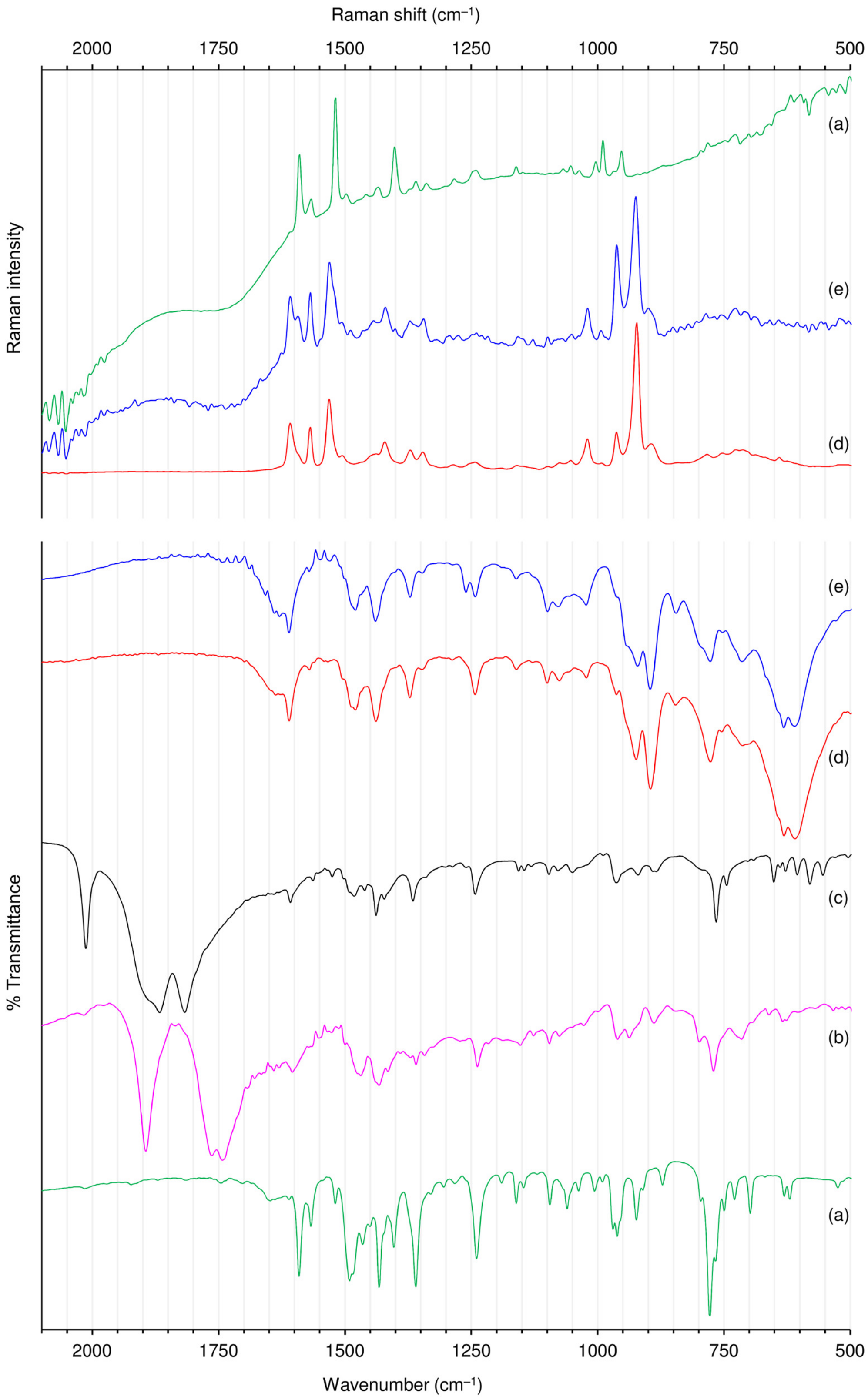

Fig. S4 FT-IR (bottom graph) and FT-Raman (top graph) spectra in the range of 500-2100 cm^{-1} of (a) [ptapzpy]Br, (b) tricarbonyl complex **1**, (c) tetracarbonyl complex **2**, and the hybrid material **3** prepared from **1** (d) and **2** (e).

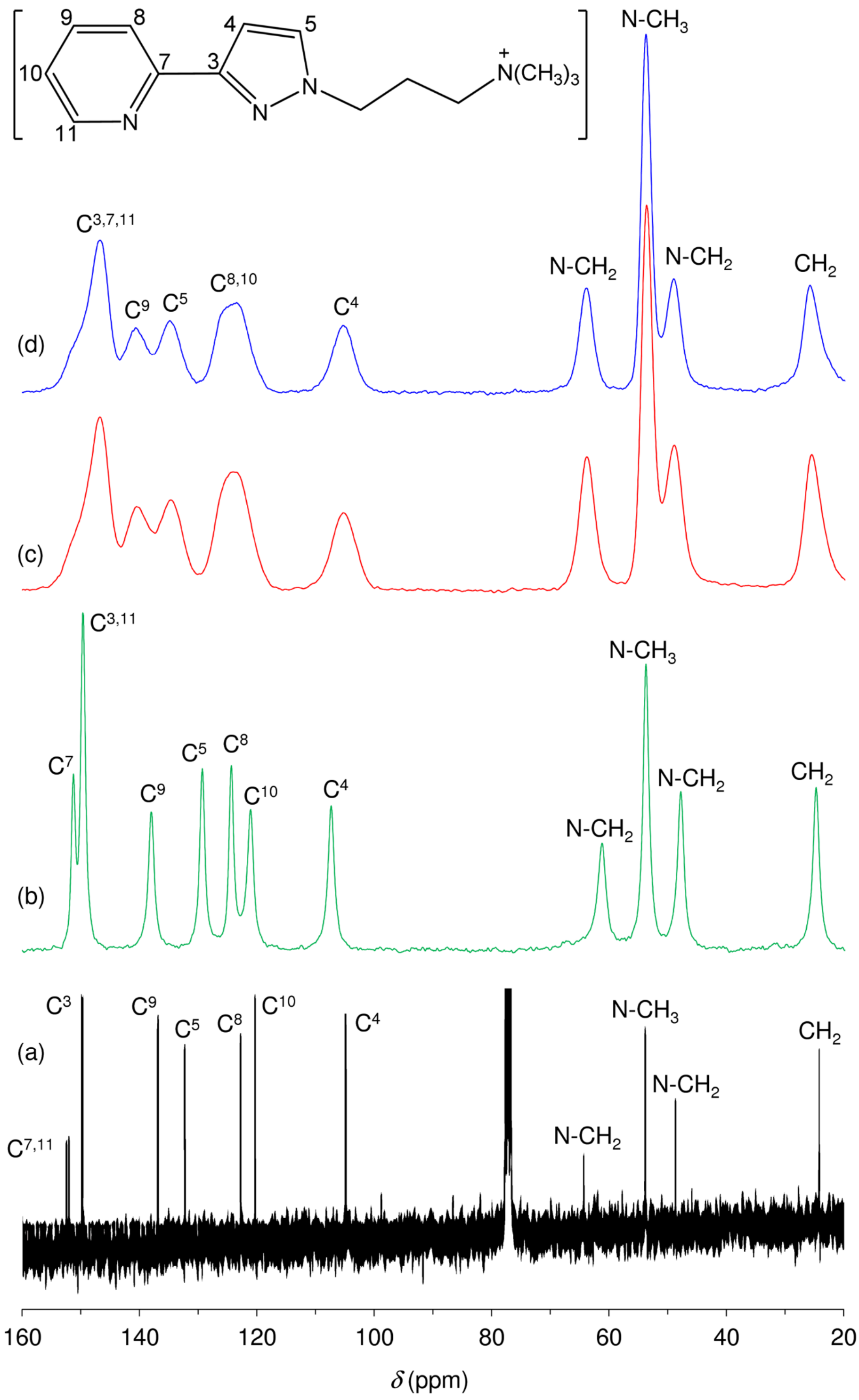

Fig. S5 ^{13}C solution (a) and $^{13}C\{^{1}H\}$ CP MAS NMR (b-d) spectra with assignments of (a,b) [ptapzpy]Br and the hybrid material **3** prepared from **1** (c) and **2** (d).

Table S3 *cis*-Cyclooctene epoxidation with $TBHP_D$ in the presence of compounds **1** and **2**, and comparison to literature data for molybdenum carbonyl complexes bearing N-donor ligands, tested as pre-catalysts, at 55 °C without additional solvent.

Entry	Complex[a]	Mo:Cy:$TBHP_D$[b]	*t* (h)	Conv. (%)[c]	Select. (%)[d]	Ref.
1	**1**	1:113:172	6/24	35/54	89/85	This work
2	**2**	1:113:172	6/24	31/52	97/92	This work
3	$[Mo(CO)_4(pzpyH)]$	1:100:152	6/24	51/78	100/100	12
4	$[Mo(CO)_4(pzpyE)]$	1:100:152	6/24	70/92	100/100	12
5	$[Mo(CO)_4(pyim)]$	1:100:153	5	100	100	13
6	$[Mo(CO)_4(PyNHC)]$	1:100:200	4/24	~3/~9	~100/~100	14
7	$[Mo(CO)_3(Py_2NHC)]$	1:100:200	4/24	~3/~16	~100/~100	14
8	$[Mo(CO)_3(Me_3\text{-tame})]$	1:100:154	24	64	100	15
9	$[Mo(\eta^3\text{-}C_3H_5)Cl(CO)_2(CH_3CN)_2]$	1:100:159	6/24	99/100	100/100	16
10	$[Mo(\eta^3\text{-}C_3H_5)Br(CO)_2(CH_3CN)_2]$	1:100:159	6/24	99/100	100/100	16
11	$[Mo(\eta^3\text{-}C_5H_5O)Br(CO)_2(CH_3CN)_2]$	1:100:159	6/24	97/100	100/100	16
12	$[Mo(\eta^3\text{-}C_3H_5)Cl(CO)_2(L^*)]$	1:100:159	6/24	88/95	100/100	16
13	$[Mo(\eta^3\text{-}C_3H_5)Cl(CO)_2(L^{**})]$	1:100:159	6/24	68/88	100/100	16
14	$[Mo(\eta^3\text{-}C_3H_5)Cl(CO)_2(L^{***})$	1:100:159	6/24	99/100	100/100	16
15	$[Mo(\eta^3\text{-}C_3H_5)Br(CO)_2(L^{***})]$	1:100:159	6/24	99/100	100/100	16
16	$[Mo(\eta^3\text{-}C_5H_5O)Br(CO)_2(L^{***})]$	1:100:159	6/24	97/100	100/100	16
17	$[CpMo(CO)_2(IMes)(CH_3CN)]BF_4$	1:100:200	4/24	~90/~100	~100/~100	17

[a] pzpyH = 2-[3(5)-pyrazolyl]pyridine; pzpyE = ethyl[3-(2-pyridyl)-1-pyrazolyl]acetate; pyim = N-(n-propyl)-2-pyridylmethanimine; PyNHC = 3-methyl-1-picolylimidazol-2-ylidene; Py_2NHC = 1,3-dipicolylimidazol-2-ylidene; Me_3-tame = 1,1,1-tris(methylaminomethyl)ethane; L* = 1,4-(2,6-dimethyl)phenyl-2,3-dimethyldiazabutadiene; L** = 1,4-(2,6-diisopropyl)-phenyldiazabutadiene; L*** = 1,4-(4-chloro)phenyl-2,3-naphthalenediazabutadiene; IMes = 1,3-bis(2,4,6-trimethylphenyl)imidazol-2-ylidene. [b] Initial Mo:Cy:$TBHP_D$ molar ratio. [c] *cis*-Cyclooctene conversion. [d] Selectivity to the epoxide (CyO).

Table S4 Comparison of FT-IR bands observed for solids recovered after catalytic reactions (entries 1-7) with corresponding bands for compounds containing the β-$[Mo_8O_{26}]^{4-}$ anion.

Compound[a]	IR bands (cm^{-1})					Ref.
**1**-S-$TBHP_D$[b]	941[c]	910[c]	840[d]	705[d]	660[d]	This work
**1**-S-$TBHP_D$-[bmim]NTf_2[b]	937	911/903	838	706/693	659	This work
**1**-S-H_2O_2[b]	940	910	841	705	658	This work
**2**-S-$TBHP_D$[b]	942	910	842	706	660	This work
**2**-S-$TBHP_D$-[bmim]NTf_2[b]	937	910/900	837	707/693	658	This work
**3**-S-$TBHP_D$[b]	941	909	841	705	659	This work
**3**-S-$TBHP_D$-[bmim]NTf_2[b]	938	911/902	839	705/691	658	This work
$(bmim)_3NH_4[Mo_8O_{26}]$ (**6**)[b]	933	909/898	837	704/692	657	This work
$(H_3biim)_4[Mo_8O_{26}]$	944	919	838	686	670	18
$(C_5H_5NH)_4[Mo_8O_{26}]$	946	912	839	709	675	19
$((CH_3)_3CC_5H_4NH)_4[Mo_8O_{26}]$	949	914	837	708	659	19
$(HDBU)_3(NH_4)[Mo_8O_{26}]\cdot H_2O$	939	910	842	720	669	20
$(Hmim)_4[Mo_8O_{26}]$	946	910	841	710	665	21
$(Dhmim)_4[Mo_8O_{26}]$	956	911	837	714	650	21
$(Hpy)_4[Mo_8O_{26}]\cdot H_2O$	947	911	836	717	632	21
$(bmim)_4[Mo_8O_{26}]$	939	913	842	714	661	3
$(BDMLPX)_3[Mo_8O_{26}]_2(H_3O)_2\cdot H_2O$	944	902	841	711	651	22
$(L)_x[\beta\text{-}Mo_8O_{26}]$[b]	935	910/898	836	699	657	23

[a] H_2biim = 2,2′-biimidazole; DBU = 1,8-diazabicyclo[5.4.0]undec-7-ene; Hmim = 1-hexyl-3-methylimidazolium; Dhmim = 1,2-dimethyl-3-hexylimidazolium; Hpy = 1-hexylpyridinium; BDMLPX = di(1,2-dimethylimidazolium) p-xylenedichloride; L = monoprotonated $[4,4'\text{-Hbipy}]^+$ cations or, alternatively, a mixture of neutral 4,4′-bipy and diprotonated $[4,4'\text{-}H_2bipy]^{2+}$ cations. [b] ATR FT-IR bands. [c] Bands attributed to $\nu(Mo{=}O_t)$ vibrations. [d] Bands attributed to ν(Mo–O–Mo) vibrations.

Table S5 Elemental analysis results.

Compound		C (%)	H (%)	N (%)
$(bmim)_3NH_4[Mo_8O_{26}]$ (**6**)	Found	17.89	2.98	6.10
$C_{24}H_{49}N_7Mo_8O_{26}$	Calcd	17.80	3.05	6.06
Recovered solid **2**-S-$TBHP_D$	Found	30.47	4.10	9.76
$(ptapzpy)_4[Mo_8O_{26}]$ ($C_{56}H_{84}N_{16}Mo_8O_{26}$) (**4**)	Calcd	31.07	3.91	10.35
Recovered solid **2**-S-$TBHP_D$-[bmim]NTf_2	Found	17.50	3.31	5.24
$(bmim)_3(H_3O)[Mo_8O_{26}]$ ($C_{24}H_{48}N_6Mo_8O_{27}$) (**5**)	Calcd	17.79	2.99	5.19

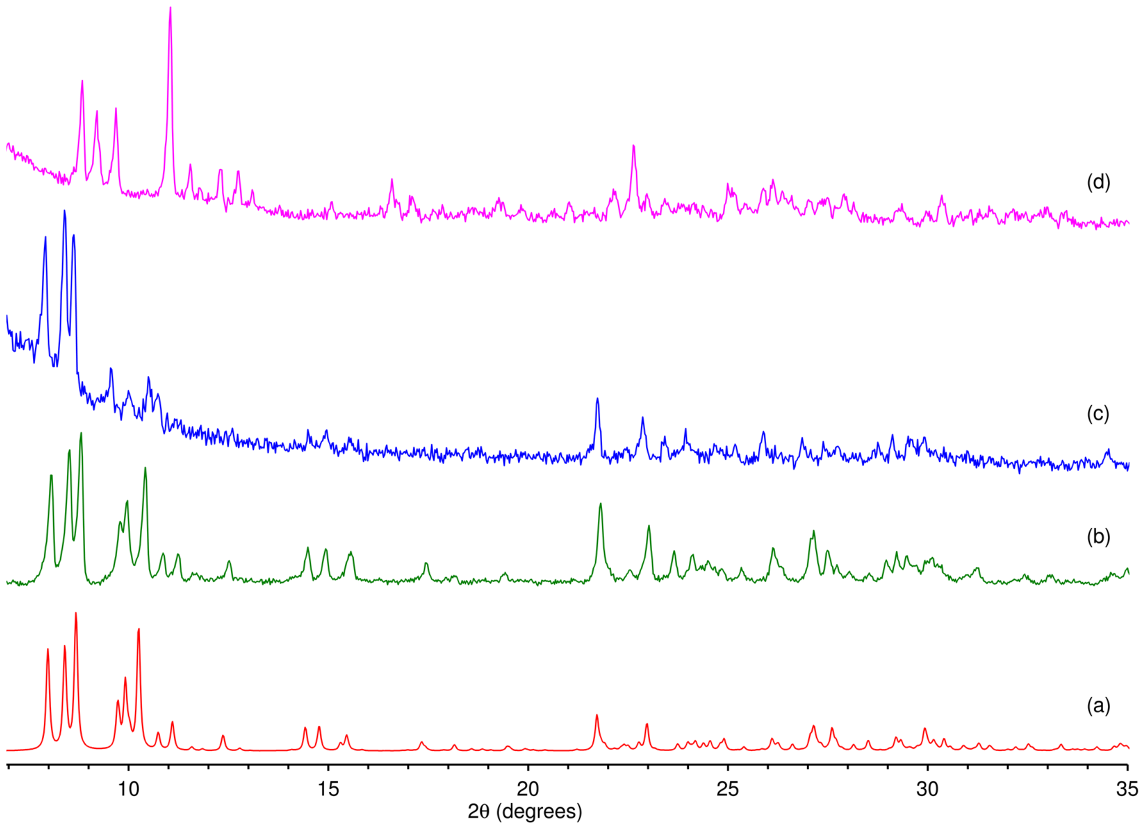

Fig. S6 Simulated (a) and experimental (b-d) powder XRD patterns of (a) $(bmim)_3NH_4[Mo_8O_{26}]$, (b) $(bmim)_3NH_4[Mo_8O_{26}]$ (**6**), (c) the recovered solid **2**-S-TBHP$_D$-[bmim]NTf$_2$, and (d) the recovered solid **2**-S-TBHP$_D$. The program Mercury (copyright CCDC, ver. 3.9)[24] was used to calculate the simulated pattern from the single-crystal X-ray data published for $(bmim)_3NH_4[Mo_8O_{26}]$.[4]

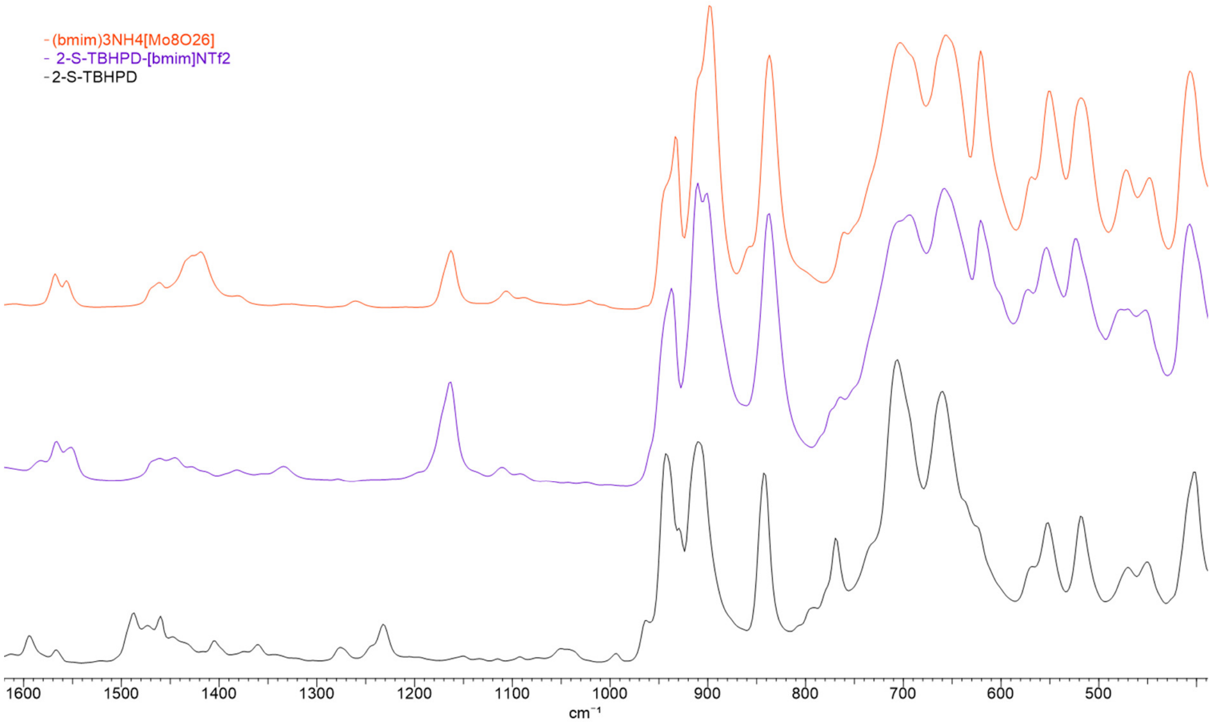

Fig. S7 ATR FT-IR spectra for the recovered solids **2**-S-TBHP$_D$ and **2**-S-TBHP$_D$-[bmim]NTf$_2$, and the salt $(bmim)_3NH_4[Mo_8O_{26}]$ (**6**).

References

1 H. Brunner and T. Scheck, *Chem. Ber.*, 1992, **125**, 701−709.

2 D. J. Darensbourg and R. L. Kump, *Inorg. Chem.*, 1978, **17**, 2680−2682.

3 C. J. Carrasco, F. Montilla, E. Álvarez, M. Herbert and A. Galindo, *Polyhedron*, 2013, **54**, 123.

4 S. L. Linguito, X. Zhang, M. Padmanabhan, A. V. Biradar, T. Xu, T. J. Emge, T. Asefa and J. Li, *New J. Chem.*, 2013, **37**, 2894.

5 T. Kottke and D. Stalke, *J. Appl. Crystallogr*., 1993, **26**, 615-619.

6 APEX2 *Data Collection Software*, Version 2012.4, Bruker AXS, Delft, The Netherlands, 2012.

7 SAINT+, *Data Integration Engine*, version 8.27b, 1997-2012, Bruker AXS, Madison, Wisconsin, USA.

8 G. M. Sheldrick, SADABS, version 2012/1, Bruker AXS Area Detector Scaling and Absorption Correction Program, Bruker AXS, Madison, Wisconsin, USA.

9 G. M. Sheldrick, *Acta Crystallogr., Sect. A: Found. Crystallogr.*, 2008, **64**, 112-122.

10 G. M. Sheldrick, SHELXT-2014, *Program for Crystal Structure Solution*, University of Göttingen, 2014.

11 G. M. Sheldrick, SHELXL, version 2014/3, *Program for Crystal Structure Refinement*, University of Göttingen, 2014.

12 P. Neves, T. R. Amarante, A. C. Gomes, A. C. Coelho, S. Gago, M. Pillinger, I. S. Gonçalves, C. M. Silva and A. A. Valente, *Appl. Catal. A: Gen.*, 2011, **395**, 71.

13 A. C. Gomes, S. M. Bruno, S. Gago, R. P. Lopes, D. A. Machado, A. P. Carminatti, A. A. Valente, M. Pillinger and I. S. Gonçalves, *J. Organomet. Chem.*, 2011, **696**, 3543.

14 Z. Wang, S. Li, W. J. Teo, Y. T. Poh, J. Zhao and T. S. A. Hor, *J. Organomet. Chem.*, 2015, **775**, 188.

15 Ž. Petrovski, A. A. Valente, M. Pillinger, A. S. Dias, S. S. Rodrigues, C. C. Romão and I. S. Gonçalves, *J. Mol. Catal. A: Chem.*, 2006, **249**, 166.

16 J. C. Alonso, P. Neves, M. J. Pires da Silva, S. Quintal, P. D. Vaz, C. Silva, A. A. Valente, P. Ferreira, M. J. Calhorda, V. Félix and M. G. B. Drew, *Organometallics*, 2007, **26**, 5548.

17 S. Li, C. W. Kee, K.-W. Huang, T. S. A. Hor and J. Zhao, *Organometallics*, 2010, **29**, 1924.

18 P. Neves, T. R. Amarante, A. A. Valente, M. Pillinger and I. S. Gonçalves, *Catal. Lett.*, 2016, **146**, 841.

19 C. A. Gamelas, P. Neves, A. C. Gomes, A. A. Valente, C. C. Romão, I. S. Gonçalves and M. Pillinger, *Catal. Lett.*, 2012, **142**, 1218.

20 V. Coue, R. Dessapt, M. Bujoli-Doeuff, M. Evain and S. Jobic, *J. Solid State Chem.*, 2006, **179**, 3615.

21 M. D. Zhou, M. J. Liu, L. L. Huang, J. Zhang, J. Y. Wang, X. B. Li, F. E. Kuhn and S. L. Zang, *Green Chem.*, 2015, **17**, 1186.

22 Y. Li, Z. Z. Shu, Q. Yang, Y. X. Zai, C. J. Ma, Z. P. Huang, Z. C. Yue and Y. Y. Niu, Synthesis and Reactivity in Inorganic Metal-Organic and Nano-Metal Chemistry, 2016, **46**, 280.

23 P. Neves, A. C. Gomes, F. A. A. Paz, A. A. Valente, I. S. Gonçalves and M. Pillinger, *Mol. Catal.*, 2017, **432**, 104.

24 C. F. Macrae, I. J. Bruno, J. A. Chisholm, P. R. Edgington, P. McCabe, E. Pidcock, L. Rodriguez-Monge, R. Taylor, J. van de Streek and P. A. Wood, Mercury CSD 2.0 - new features for the visualization and investigation of crystal structures, *J. Appl. Crystallogr.*, 2008, **41**, 466.
